# Social norms and obesity prevalence: From cohort to system dynamics models

**DOI:** 10.1111/obr.13044

**Published:** 2020-05-13

**Authors:** Loes Crielaard, Pritha Dutta, Rick Quax, Mary Nicolaou, Nadège Merabet, Karien Stronks, Peter M.A. Sloot

**Affiliations:** ^1^ Department of Public Health Amsterdam UMC, University of Amsterdam, Amsterdam Public Health Research Institute Amsterdam The Netherlands; ^2^ Institute for Advanced Study University of Amsterdam Amsterdam The Netherlands; ^3^ Complexity Institute Nanyang Technological University Singapore; ^4^ Interdisciplinary Graduate Programme Nanyang Technological University Singapore; ^5^ Computational Science Lab University of Amsterdam Amsterdam The Netherlands

**Keywords:** body weight perception, obesity, social norms, system dynamics modelling

## Abstract

Group‐level obesity can be seen as an emergent property of a complex system, consisting of feedback loops between individual body weight perception, individual weight‐related behaviour and group‐level social norms (a product of group‐level ‘normal' body mass index (BMI) and sociocultural ‘ideal' BMI). As overweight becomes normal, the norm might be counteracting health awareness in shaping individual weight‐related behaviour. System dynamics modelling facilitates understanding and simulating this system's emergent behaviour. We constructed six system dynamics models (SDMs) based on an expert‐informed causal loop diagram and data from six sociocultural groups (Dutch, Moroccan and South‐Asian Surinamese men and women). The SDMs served to explore the effect of three scenarios on group‐level BMI: ‘what if' weight‐related behaviour were driven by (1) health awareness, (2) norms or (3) a combination of the two. Median BMI decreased approximately 50% and 30% less in scenarios 2 and 3, respectively, than in 1. In men, the drop in BMI was approximately two times larger in scenario 1 versus 3, whereas in women, the drop was approximately equal in these scenarios. This study indicates that the overweight norm in men holds group‐level BMI close to overweight despite health awareness. Since norms are counteracting health awareness less strongly in women, other drivers of obesity must be more relevant.

**Abbreviations**
BMI: body mass index
BMR: basal metabolic rate
CLD: causal loop diagram
PAL: physical activity level
SDM: system dynamics model
TDEE: total daily energy expenditure
TDEI: total daily energy intake

## 1 BACKGROUND

It has been argued that individuals are more likely to underestimate their own body weight as obesity prevalence increases.^1^ That is because body weight perception, a determinant of overweight and obesity,^2^ is partly determined via social comparison:^3^ the typical weight at the group level influences how individuals evaluate their own weight.^1^ Body weight perception is thus affected by what is normal. It however also affects what is normal via its impact on obesity prevalence, inducing feedback loops that may over time contribute to group‐level obesity. These feedback loops result in complex behaviour, possibly inducing group‐level obesity as an emergent property. An emergent property of a complex system cannot plainly be derived from its parts and is more than a direct outcome of their aggregation.^4^ In addition, a complex system shows non‐linear behaviour, with a large disproportionality between its inputs and outputs,^5^ which often results from the presence of feedback loops.

Previous research has indicated that norms affect individual dietary intake and physical activity. It has been shown that it is problematic for individuals to change their weight‐related behaviour if others in their environment do not.^6^ In fact, it has consistently been shown that weight loss resulting from interventions targeting individual weight‐related behaviour is likely to not be maintained.^7^ One possible explanation is that norms may counteract health awareness in shaping individual health‐related behaviour. In that regard, the relevance of addressing norms to change individual health‐related behaviour as a policy strategy has received attention with respect to smoking.^8^ That is, ‘the shifts in social norms (…) that occur as a result of major tobacco control interventions and campaigns can strengthen smokers' motivation to quit and commitment to staying quit'.^9^ Understanding the effect of the feedback loops between individual characteristics and group‐level processes over time can guide similar policy strategies aiming to lower obesity prevalence, in which we do justice to influences at both levels.

System dynamics models (SDMs) can aid in understanding and simulating this system's emergent behaviour.^10^ SDMs can reveal how variables interact by expressing the causal links between them using difference equations. Specifically, system dynamics modelling is used to understand and simulate a complex system's non‐linear behaviour in different scenarios.^10^ This is valuable when evaluating the effect of ‘what if' scenarios is unfeasible using conventional empirical methods, as holds for comparing the effect of health awareness versus norms on group‐level body mass index (BMI).

In this study, we model the system of social norms regarding body weight perception and obesity prevalence using SDMs. These SDMs are designed to test the hypothesis that as overweight becomes normal, the norm might be counteracting health awareness in shaping individual weight‐related behaviour. Hereto, we design an expert‐informed causal loop diagram (CLD) to conceptualize this system, which we subsequently use as a template for functioning SDMs. We consider this system as consisting of feedback loops between individual body weight perception, individual weight‐related behaviour and group‐level norms towards body weight. These norms can be conceptualized as a product of social comparison and cultural preferences:^3^ what is considered normal, i.e. group‐level BMI, and ideal, i.e. sociocultural ideal BMI. The SDMs simulate this system's behaviour for a multi‐ethnic cohort in Amsterdam (registered in the HELIUS study)^11^—for whom body weight perception was measured and whose data we use to tune the SDMs.

We present this—as a proof‐of‐concept of studying feedback loops between individual characteristics and group‐level processes using this methodology—with the aim to simulate the effect of three scenarios on group‐level BMI. These reflect the question ‘what if' weight‐related behaviour were driven (1) only by health awareness, (2) only by norms and (3) by their interaction, i.e. health awareness and norms.

## 2 METHODS

In this section, we describe (1) the formulation of the expert‐informed CLD, (2) the study population that we select from the Amsterdam‐based cohort whose data can be used to quantify this CLD and (3) body weight perception as it was measured for this study population. We then explain (4) the conversion of the CLD to six SDMs using stocks, flows, auxiliaries and constants and (5) the variables and equations that we use in these SDMs. Lastly, we describe (6) the use of cross‐sectional data for the quantification of the SDMs, (7) the use of validation statements to validate this quantification and (8) the scenarios we test using the validated SDMs.

### 2.1 Causal loop diagram

**FIGURE 1:**
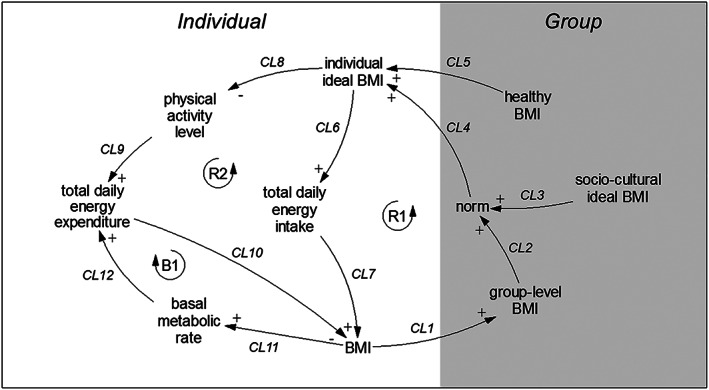
Causal loop diagram (CLD) of the system of social norms regarding body weight perception and obesity prevalence. Variables are connected by arrows indicating causal links, where a plus indicates that an increase in the variable at the tail of the arrow constitutes an increase in the variable at the head of the arrow whereas a minus indicates that an increase in the variable at the tail constitutes a decrease in the variable at the head. Reinforcing feedback loops R1 and R2 and balancing feedback loop B1 are indicated with loop symbols

We start by formulating an expert‐informed CLD of the system, through iteratively conducting interviews, facilitating the integration of expert knowledge concerning public health, healthy inequalities, dietary behaviour, sociology and anthropology. The resulting diagram (Figure 1) shows these experts' understanding of the system, depicting its variables and causal links. Subsequently, we use literature to confirm and support each individual causal link proposed by the experts.

The CLD shows that an increase in individual BMI increases group‐level BMI (CL1),^3^ that is, what is normal, which in turn drives up the norm (CL2).^3^ Sociocultural ideal BMI, that is, what is ideal, also affects the norm (CL3)^3^ and represents the relatively stable sociocultural perception an individual has of the ideal BMI in their group, which induces an individual variation in how the norm is regarded. Individual ideal BMI, representing the BMI an individual strives for, is then driven both by the norm (CL4)^12^ and by knowledge of what a healthy BMI is (CL5),^12^ creating a possible conflict between these two influences. This sequence leading to individual ideal BMI drives two feedback loops in parallel (R1 and R2). In R1, an increase in individual ideal BMI increases food intake (total daily energy intake (TDEI)) (CL6),^13^, ^14^, ^15^, ^16^ which then increases BMI (CL7).^17^ In R2, this increase in individual ideal BMI causes a decrease in physical activity level (PAL) (CL8)^14^, ^18^, ^19^ and consequently in total daily energy expenditure (TDEE) (CL9),^20^ which similarly increases BMI (CL10).^17^ TDEE is also influenced by basal metabolic rate (BMR) (CL12),^21^ which increases as BMI increases (CL11),^21^ causing a balancing feedback loop (B1).

### 2.2 Study population

We then convert the CLD to SDMs, which requires data that can quantitatively represent the CLD's variables. Therefore, we turn to the multi‐ethnic HELIUS study,^11^ in which data on characteristics reflecting these variables were collected.

We construct six SDMs for three ethnic groups from this cohort, as we hypothesize that norms influence individuals to a different extent in cultures that contrast concerning the degree of individualism/collectivism. We define the positions of these cultures on the dimension of individualism/collectivism as previously identified based on the Hofstede model, a well‐established paradigm for comparing cultures.^22^ Here, collectivism, versus its opposite individualism, is ‘the degree to which people in a society are integrated into groups'.^22^ We accordingly construct SDMs for three groups that differ in the degree of individualism/collectivism, which we then stratify by gender—as we expect the influence of norms on women to be greater^23^—resulting in six groups. These are Dutch (‘individualistic'),^24^ Moroccan (‘collectivistic')^24^ and South‐Asian Surinamese (Suriname—‘slightly collectivistic'^24^; South‐Asian ancestry from India^25^—‘both collectivistic and individualistic traits')^24^ men and women. We confirm the diversity in body weight perception that underlies this stratification with statistical analyses (Appendix S1.1). Constructing an SDM for each of these groups implies that we assume that, specifically for social comparisons of body weight, a member of one group is unaffected by the individuals in other groups. This assumption is based on the similarity hypothesis, which is the idea that ‘comparison with targets that are close on a variety of dimensions, such as age, gender, or family ties, have a greater affective impact'.^3^ Thus, we expect that individuals are most likely to compare themselves to others from their own sociocultural group.

The data include characteristics concerning sex (which we use as a proxy for gender), age, ethnicity, education, BMI and body weight perception for each individual. Details on their collection are given in Appendix S1.1. In each of the groups, we select only those individuals who have ‘lower vocational schooling or lower secondary schooling' or ‘intermediate vocational schooling or intermediate/higher secondary schooling'^11^ as the highest educational level obtained. Selecting only certain education segments allows us to disregard educational differences when analysing the results. This results in 5,299 participants (Dutch men: *n* = 753; Moroccan men: *n* = 774; South‐Asian Surinamese men: *n* = 839; Dutch women: *n* = 848; Moroccan women: *n* = 1,086; South‐Asian Surinamese women: *n* = 999).

### 2.3 Body weight perception

**FIGURE 2:**
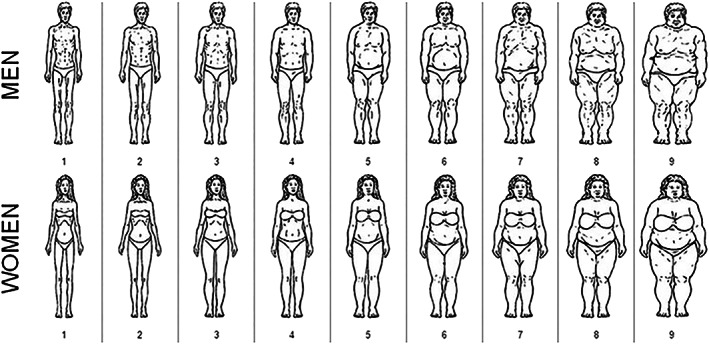
Body image scale

For this cohort, perceived BMI was determined via questionnaires, asking each individual to indicate which image they most looked like on a randomly ordered version of the body image scale developed by Pulvers et al.^26^ (Figure 2).

For all groups we map each image to a corresponding measured BMI, where each image is represented by the average BMI for all individuals that selected that image as their perceived BMI (Table 1).

**TABLE 1:** Perceived body mass index (BMI) (body image scale) in kg/m^2^ corresponding to average measured BMI per group

Image	Dutch men (*n* = 753)	Moroccan men (*n* = 774)	South‐Asian Surinamese men (*n* = 839)	Dutch women (*n* = 848)	Moroccan women (*n* = 1,086)	South‐Asian Surinamese women (*n* = 999)
1	20.1 (*n =* 10)	19.3 (*n =* 14)	20.8 (*n =* 22)	18.5 (*n =* 9)	18.6 (*n =* 25)	20.4 (*n =* 18)
2	22.0 (*n =* 70)	21.8 (*n =* 61)	20.9 (*n =* 74)	20.2 (*n =* 76)	20.3 (*n =* 89)	21.1 (*n =* 75)
3	23.0 (*n =* 146)	23.6 (*n =* 157)	23.2 (*n =* 151)	22.2 (*n =* 202)	22.8 (*n =* 215)	22.5 (*n =* 172)
4	25.4 (*n =* 234)	26.0 (*n =* 254)	25.3 (*n =* 230)	25.0 (*n =* 263)	25.7 (*n =* 293)	25.1 (*n =* 236)
5	28.4 (*n =* 243)	29.1 (*n =* 227)	27.8 (*n =* 299)	28.1 (*n =* 168)	28.4 (*n =* 242)	27.8 (*n =* 240)
6	32.2 (*n =* 36)	31.8 (*n =* 38)	31.2 (*n =* 38)	31.5 (*n =* 71)	31.6 (*n =* 114)	30.9 (*n =* 132)
7	33.0 (*n =* 10)	34.1 (*n =* 15)	31.7 (*n =* 16)	34.2 (*n =* 40)	34.0 (*n =* 67)	32.0 (*n =* 69)
8	38.0 (*n =* 2)	33.3 (*n =* 6)	34.8 (*n =* 7)	38.0 (*n =* 14)	37.7 (*n =* 33)	34.2 (*n =* 44)
9	40.2 (*n =* 2)	36.3 (*n =* 2)	41.9 (*n =* 2)	41.7 (*n =* 5)	39.8 (*n =* 8)	40.0 (*n =* 13)

In addition, each individual was asked which image they would prefer to look like and which image they thought others in their environment would find most attractive. We used the answers to these two questions to quantitatively represent individual ideal BMI and sociocultural ideal BMI, respectively, based on the abovementioned calibration (visual representation in Appendix S1.2).

### 2.4 Stocks, flows, auxiliaries and constants

Using these data, we then convert the CLD into six SDMs. The structure of the SDMs is considered representative for each of the groups, but quantification of the strengths of the causal links for each SDM is dependent on each group's data. From here onwards, the conceptual variables in the CLD are defined as quantifiable variables based on the cohort data (see Section 2.5).

**FIGURE 3:**
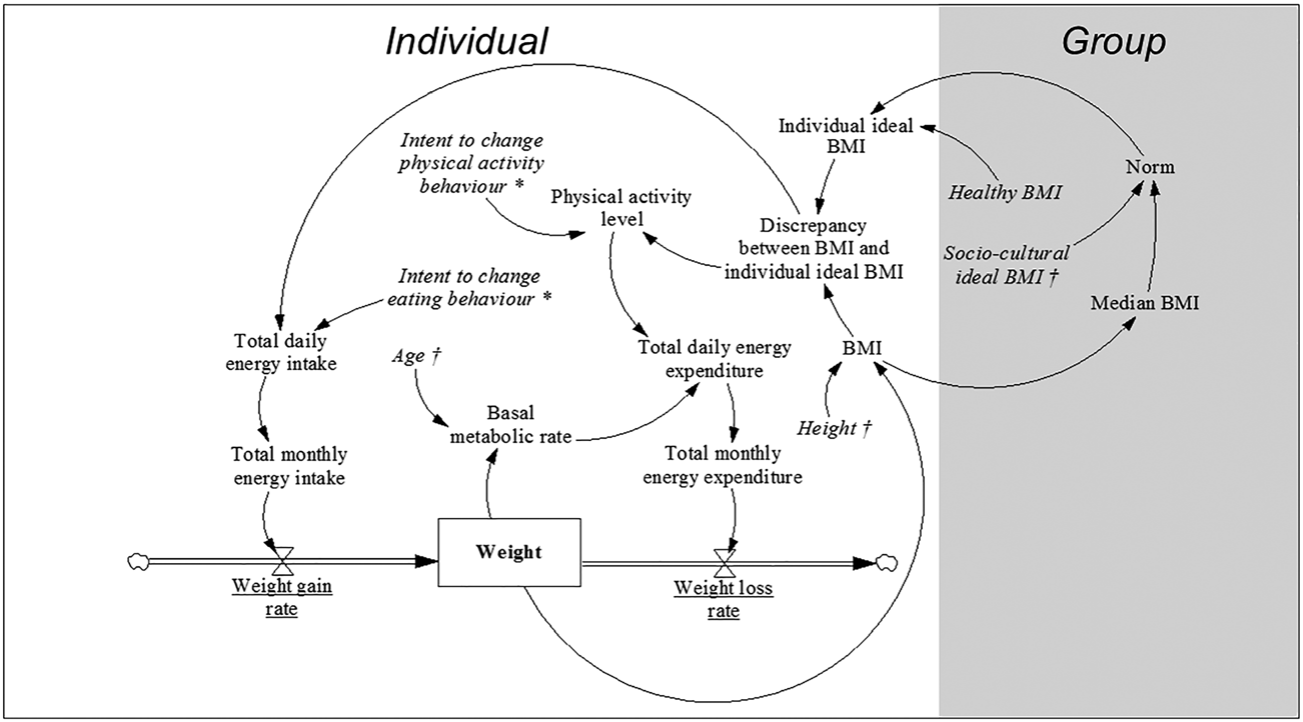
System dynamics model (SDM) mirroring the expert‐informed causal loop diagram (CLD) of the system of social norms regarding body weight perception and obesity prevalence. Variables (see Section 2.5) are connected by arrows indicating causal links. The stock is shown as a box (variable name displayed in bold, i.e. ‘Weight'), whereas flows are displayed as thick arrows (regulated by valves, variable names are underlined). Auxiliaries and constants are indicated in regular font and italics, respectively. Optimized parameters and constants based on initial values from the cohort data are marked with ^*^ and ^†^, respectively

The SDMs' structure (Figure 3) is designed to mirror the CLD but contains a number of additional variables (e.g. ‘Age' and ‘Height') that are necessary to meet the mathematical and structural SDM requirements. These requirements have been described in detail elsewhere.^27^, ^28^, ^29^, ^30^, ^31^ To facilitate the conversion of the CLD into six SDMs, we define which of the CLD's variables will be stocks, flows, auxiliaries and constants. A stock represents a ‘concrete aspect […] of the system that can be seen and measured' (‘Weight' in Figure 3), whereas a flow is connected to a stock and determines how it changes over time—it can be perceived as a rate (e.g. ‘Weight loss rate').^31^ An auxiliary is ‘any dynamic variable that is computed from other variables at a given time' (e.g. ‘BMI'), whereas a constant is a variable that does not change during the simulation (e.g., ‘Height').^32^

### 2.5 Variables and equations

The variables are formulated based on the cohort data (Table 2) and are displayed in *italics* in the remainder of Section 2.

**TABLE 2:** Description of variables used in the system dynamics models (SDMs)

	Variable	Abbreviation	Units	Type
Anthropometric and demographic variables	Gender	‐	‐	‐
	Ethnicity	‐	‐	‐
	Weight	*W*	kg	Stock
	Height	*H*	m	Constant (initial value)
	BMI	*BMI*	kg/m^2^	Auxiliary
	Median BMI	*MedBMI*	kg/m^2^	Auxiliary
	Age	*Age*	years	Constant (initial value)
Body weight perception variables	Individual ideal BMI	*IIB*	kg/m^2^	Auxiliary
	Sociocultural ideal BMI	*SCIB*	kg/m^2^	Constant (initial value)
	Norm	*Norm*	kg/m^2^	Auxiliary
	Discrepancy between BMI and individual ideal BMI	*Discrepancy*_*BMIandIIB*_	kg/m^2^	Auxiliary
	Healthy BMI	*HB*	kg/m^2^	Constant
Energy balance variables	Physical activity level	*PAL*	‐	Auxiliary
	Total daily energy intake	*TDEI*	kcal/day	Auxiliary
	Total monthly energy intake	*TMEI*	kcal/month	Auxiliary
	Basal metabolic rate	*BMR*	kcal/day	Auxiliary
	Total daily energy expenditure	*TDEE*	kcal/day	Auxiliary
	Total monthly energy expenditure	*TMEE*	kcal/month	Auxiliary
	Weight gain rate	*Rate*_*Weight gain*_	kg/month	Flow
	Weight loss rate	*Rate*_*Weight loss*_	kg/month	Flow
Optimized parameters	Intent to change physical activity behaviour	*Intent*_*PAB*_	1/(kg/m^2^)	Constant
	Intent to change eating behaviour	*Intent*_*EB*_	(kcal/day)/(kg/m^2^)	Constant
Fitted parameter	Impact healthy BMI on individual ideal BMI	*Impact*_*HBonIIB*_	‐	Constant

*Note*: Gender and ethnicity are not technically variables included in the SDMs but are shown as we use them in the development of the SDMs. The values for *MedBMI*, the optimized parameters and the fitted parameter are the same for all individuals in a given group (as explained in this section and in Section 2.6). We treat *Age* as a constant. *PAL* has no unit: It is expressed as *TDEE/BMR* (see Appendix S1.3).^21^ *Impact*_*HBonIIB*_ also has no unit, it is expressed as *BMI/BMI* (as explained in this section).

We use a list of equations (Table 3), representing the SDMs' structure, that takes the values corresponding to all variables in Table 2 for a given individual as inputs and generates their values for the next time point as outputs. These equations should be solved in the order as defined in Figure 3 (starting from *IIB* (individual ideal BMI) and following the causal links).

**TABLE 3:** The list of equations that represents the system dynamics models (SDMs)

Equation					Causal link
(1)	*IIB*_*t*_ = *Impact*_*HBonIIB*_ × *HB*+(1 − *Impact*_*HBonIIB*_) × *Norm*_*t*_				CL4; CL5
(2)	*Discrepancy*_*BMIandIIBt*_ = *BMI*_*t*_ − *IIB*_*t*_				
(3)	*PAL*_*t*+1_ = *PAL*_*t* = 0_+*Intent*_*PAB*_ × *Discrepancy*_*BMIandIIBt*_				CL8
(4)	*TDEI*_*t*+1_ = *TDEI*_*t* = 0_+*Intent*_*EB*_ × *Discrepancy*_*BMIandIIBt*_				CL6
(5)	*TDEE*_*t*+1_ = *BMR*_*t*_ × *PAL*_*t*+1_				CL9; CL12
(6)	$W_{t+1}=W_{t}+\left({\text{Rate}}_{\text{Weight gain}}-{\text{Rate}}_{\text{Weight loss}}\right)=W_{t}+\left(\frac{{\text{TMEI}}_{t+1}}{7700}-\frac{{\text{TMEE}}_{t+1}}{7700}\right)=W_{t}+\left(\frac{{\text{TDEI}}_{t+1}}{7700}\times \frac{365}{12}-\frac{{\text{TDEE}}_{t+1}}{7700}\times \frac{365}{12}\right)=W_{t}+\left(\frac{73\times {\text{TDEI}}_{t+1}}{18480}-\frac{73\times {\text{TDEE}}_{t+1}}{18480}\right)$				CL7; CL10
(7)	${\mathrm{BMI}}_{t+1}=\frac{W_{t+1}}{{H_{t=0}}^{2}}$				
(8)	*BMR*_*t*+1_	Men	*Age*_*t* = 0_ 18–30	*BMR*_*t*+1_ = 15.057 × *W*_*t*+1_+692.2	CL11
			*Age*_*t* = 0_ 30–60	*BMR*_*t*+1_ = 11.472 × *W*_*t*+1_+873.1	
			*Age*_*t* = 0_ ≥60	*BMR*_*t*+1_ = 11.711 × *W*_*t*+1_+587.7	
		Women	*Age*_*t* = 0_ 18–30	*BMR*_*t*+1_ = 14.818 × *W*_*t*+1_+486.6	
			*Age*_*t* = 0_ 30–60	*BMR*_*t*+1_ = 8.126 × *W*_*t*+1_+845.6	
			*Age*_*t* = 0_ ≥60	*BMR*_*t*+1_ = 9.082 × *W*_*t*+1_+658.5	
(9)	*MedBMI*_*t*+1_ = *Median*(*BMI*_*t*+1_)				CL1
(10)	${\text{Norm}}_{t+1}=\frac{{\text{MedianBMI}}_{t+1}+{\text{SCIB}}_{t=0}}{2}$				CL2; CL3

*Note*: We show how the equations are connected to the causal links as specified in the causal loop diagram (CLD) (Figure 3) on the right.

These equations ensure that dimensional consistency is preserved within the SDMs, that is, the units between the left‐ and the right‐hand side of all equations are consistent.^27^, ^28^, ^29^, ^30^, ^31^

The body weight perception variables and their updating procedures and the fitted parameter (Equations 1, 2 and 10 as listed in Table 3) are described below. The optimized parameters and their function (Equations 3 and 4) are described in Section 2.6. The remaining variables and their updating procedures are described in Appendix S1.3 as they were previously defined and validated elsewhere.

The updating procedure for *IIB* takes *HB* (healthy BMI), *Norm* and *Impact*_*HBonIIB*_ (impact healthy BMI on individual ideal BMI) into account (Equation 1). We define *HB* as a constant that equals 22.5 kg/m^2^ for all groups, representing a conservative estimate of a healthy BMI and corresponding to the cut‐off point for an elevated type 2 diabetes risk.^33^, ^34^ The updating procedure for *Discrepancy*_*BMIandIIB*_ (discrepancy between BMI and individual ideal BMI) is then defined by the difference between *BMI* and *IIB* at a particular time step, which describes the gap between the BMI an individual has and the BMI they would like to attain (Equation 2).

We define *Norm* as the average between a group's *MedBMI* (median BMI), representing group‐level BMI, and the respective individual's initial value for *SCIB* (sociocultural ideal BMI) (Equation 10). We choose the median here, as opposed to the average, because we hypothesize that an individual might be inclined to alter their BMI based on the frequency of individuals in their group with a different BMI. The average is considered unsuitable as it would also be affected by a relatively small fraction of the group with a different BMI, which we do not suppose would change the behaviour of most individuals. We use the initial value for *SCIB* as a constant, which via the calibration explained in Section 2.3 could be derived from the cohort data. We use this initial value for *SCIB* as a constant to introduce individual variation to the norm, as it represents the sociocultural perception an individual has of the ideal BMI in their group, which we consider to be relatively stable. Note that we choose to let both *MedBMI* and *SCIB* exert an equal influence on *Norm*, that is, the most conservative ratio of 50/50, as we are unaware of research that has specifically addressed this issue.

We form Equation 1 by rewriting the output of regression through the origin between distance *Norm* and *HB* (the independent variable, *x*), represented by

x=Norm−HB,

and distance *Norm* and *IIB* (the dependent variable, *y*), that is,

y=Norm−IIB,

which we conduct for each group. This provides us with the value for *Impact*_*HBonIIB*_ for each group, given by

$$\text{Norm}-\mathrm{IIB}={\text{Impact}}_{\text{HBonIIB}}\times \left(\text{Norm}-\mathrm{HB}\right),$$

which we can rewrite to get Equation 1. We thus determine the value for *Impact*_*HBonIIB*_ directly from the cohort data, where the interpretation of Equation 1 is that *IIB* is a weighted average of *HB* and *Norm* where *Impact*_*HBonIIB*_ determines the relative weight of each. A larger *Impact*_*HBonIIB*_ implies a larger effect of *HB* on *IIB* and, as a consequence, a lower effect of *Norm* on *IIB.* This is based on the assumptions that (1) if *Norm* equals *HB*, then *IIB* equals *HB*; implying individuals prefer to be healthy and (2) *HB* and *Norm* work against each other. The values for *Impact*_*HBonIIB*_ for each group are given in Table 4.

**TABLE 4:** Values for *Impact*_*HBonIIB*_ for each group

Sociocultural group	*Impact*_*HBonIIB*_
Dutch men	0.21
Moroccan men	0.18
South‐Asian Surinamese men	0.27
Dutch women	0.37
Moroccan women	0.47
South‐Asian Surinamese women	0.51

*Impact*_*HBonIIB*_ can also be regarded as uniquely determining an individual's behaviour with respect to *HB* and *Norm*, which we show in Appendix S1.3.

Descriptive characteristics of the study population at *t* = 0 corresponding to the SDMs' variables are presented in Table 5.

**TABLE 5:** Descriptive characteristics of the study population at *t* = 0, where we give median values for all descriptives in accordance with the use of *MedBMI* to represent group‐level BMI

		Dutch men (*n* = 753)	Moroccan men (*n* = 774)	South‐Asian Surinamese men (*n* = 839)	Dutch women (*n* = 848)	Moroccan women (*n* = 1,086)	South‐Asian Surinamese women (*n* = 999)
*Age* (years), median (IQR)		52 (24)	39 (18)	46 (23)	51 (20)	33 (18)	48 (18)
*BMI* (kg/m^2^), median (IQR)		25.6 (5.0)	26.2 (5.2)	25.4 (4.5)	24.8 (6.3)	26.1 (6.9)	26.0 (6.6)
*BMI* ≥ 25.0 kg/m^2^ (%)		57.2	62.7	55.5	48.2	59.4	57.6
*IIB* (kg/m^2^), median (IQR); mean ± SD^a^		25.4 (2.4); 24.6 **±** 1.8	26.0 (2.3); 25.3 **±** 1.6	25.3 (2.1); 24.5 **±** 1.6	22.2 (2.8); 23.0 **±** 2.3	22.8 (3.0); 23.7 **±** 2.4	22.5 (2.6); 23.6 **±** 2.3
*SCIB* (kg/m^2^), median (IQR); mean ± SD^a^		25.4 (2.4); 24.7 **±** 1.8	26.0 (2.3); 25.4 **±** 2.0	25.3 (2.1); 24.7 **±** 2.2	22.2 (2.8); 22.9 **±** 2.3	22.8 (3.0); 23.8 **±** 2.9	22.5 (2.6); 23.6 **±** 2.7
*Norm* (kg/m^2^), median (IQR)		25.5 (1.2)	26.1 (1.2)	25.4 (1.0)	23.5 (1.4)	24.4 (1.5)	24.2 (1.3)
Discrepancy between perceived BMI and *IIB* (kg/m^2^)^b^	<0 (%)	12.2	18.7	14.1	3.1	9.5	4.0
	=0 (%)	38.8	34.6	34.2	31.6	25.0	24.9
	>0 (%)	49.0	46.6	51.7	65.3	65.6	71.1
*PAL*	1.6 (%)	27.5	45.0	41.1	24.4	58.5	49.3
	1.7 (%)	72.5	55.0	58.9	75.6	41.6	50.8
*TDEI* (kcal), median (IQR)		3,002 (448)	2,981 (379)	2,854 (398)	2,354 (297)	2,332 (308)	2,251 (273)
*BMR* (kcal), median (IQR)		1,797 (267)	1,800 (216)	1,727 (229)	1,402 (176)	1,422 (174)	1,370 (162)

a We provide both the median and the mean for *IIB* and *SCIB* as providing the median only for these variables makes them appear identical.

b Here, discrepancy is calculated between perceived BMI (obtained by calibration of measured BMI to the body image scale) and *IIB* so that both are estimated according to the body image scale to allow for direct comparison. Therefore, if discrepancy < 0, individuals think they should gain weight; if discrepancy = 0, individuals are satisfied with their weight; if discrepancy > 0, individuals think they should lose weight.

### 2.6 Cross‐sectional data

The data we have are cross‐sectional; however, to construct operational SDMs, we require data from multiple time points. We therefore develop a new way to generate pseudo‐time series data from the available cross‐sectional data by generating a set of qualitative ‘data‐generating assumptions'. A summary of alternative methods and a detailed explanation of our method are given in Appendix S1.4. These assumptions are based on the system's temporal behaviour that is expected to exist across all groups, for example, that, on average, an individual can lose 2 kg/month.^35^, ^36^ Here, we assume linear dynamics for the system's short‐term behaviour. We fit the time steps in the SDMs as months in this study to identify an approximate timescale of the system's behaviour. However, this timescale is not exact, and therefore, the relative trends are more important to interpret than the exact timescale they occur on.

We optimize the values for the parameters *Intent*_*PAB*_ and *Intent*_*EB*_ such that the SDMs reproduce the data‐generating assumptions and fit the cohort data. *Intent*_*PAB*_ and *Intent*_*EB*_ represent the change that individuals make in physical activity behaviour (changing their *PAL*) and eating behaviour (changing their *TDEI*), respectively, based on how much their *BMI* differs from their *IIB* (captured in *Discrepancy*_*BMIandIIB*_). We use the optimization method basin‐hopping to find the optimal values for these parameters.^37^, ^38^ We generate six sets of two optimized parameter values: one set for each group. The parameter values are optimized using a cost function that was designed to include a mathematical representation of each of the data‐generating assumptions. This cost function enables us to introduce a temporal aspect to the available cross‐sectional data by constraining the parameter space for *Intent*_*PAB*_ and *Intent*_*EB*_ to include only SDMs which satisfy the data‐generating assumptions. That is, the cost function can be thought of as implicitly placing a next time point in the data for each individual (resulting in a so‐called pseudo‐time series) and then evaluating how far off a given SDM is from reproducing this second data point. These optimized parameter values thus correspond to the SDM that best fits the cohort data for that group in conjunction with the data‐generating assumptions.

### 2.7 Validation statements

We then validate the SDMs using a number of validation statements. Formal model validation for SDMs can be divided into two stages: structural validation and behaviour validation.^39^ Structural validity refers to whether the internal structure of the SDMs accurately describes the components of the system that are relevant to the phenomenon, whereas behaviour validity refers to whether the behaviour of the SDMs adequately reproduces real behaviour.^39^ Behaviour validation requires longitudinal data, that is, data that enable us to validate the behaviour patterns over time. We therefore only address structural validation, which consists of two components: tests addressing direct structure and structure‐oriented behaviour. Direct structure tests ‘assess the validity of the model structure, by direct comparison with knowledge about real system structure', without simulation, which ‘involves taking each relationship (mathematical equation or any form of logical relationship) individually and comparing it with available knowledge about the real system'.^39^ We address direct structure by basing the CLD on literature, using equations that were previously defined and validated elsewhere where possible and maintaining dimensional consistency in the SDMs.

As structure‐oriented behaviour tests, which ‘assess the validity of the structure indirectly, by applying certain behaviour tests on model‐generated behavior patterns' using simulation,^39^ we generate 32 qualitative validation statements derived from expert knowledge and literature (see Appendix S1.5 for references and further details). The majority of the literature behind the statements is based on previous empirical research that addresses the Dutch population in particular, which adds to the internal validity of the statements. These statements contain comparisons we make concerning how the SDMs' behaviours are expected to differ among the groups. We hypothesize for instance that norms tend to have a larger effect in the Moroccan than the Dutch, as Moroccan culture is regarded as being more collectivistic than Dutch culture.^24^ We expect a representative set of SDMs to reproduce a considerable number of these validation statements. A set can obtain a validation score out of 32 points (one for each statement), representing its quality.

### 2.8 Scenarios

The validated SDMs are subsequently used to simulate the effect of health awareness versus norms on group‐level median BMI. We simulate the effect of three scenarios on group‐level BMI in all six groups: ‘what if' weight‐related behaviour were driven (1) only by health awareness, (2) only by norms and (3) by health awareness and norms combined.

For driven only by health awareness, we set *Impact*_*HBonIIB*_ to 1 in Equation 1, making *IIB* only dependent on (equal to) *HB*:

$${\mathrm{IIB}}_{t}={\text{Impact}}_{\text{HBonIIB}}\times \mathrm{HB}+\left(1-{\text{Impact}}_{\text{HBonIIB}}\right)\times {\text{Norm}}_{t},$$

$${\mathrm{IIB}}_{t}=1\times \mathrm{HB}+\left(1-1\right)\times {\text{Norm}}_{t},$$

$${\mathrm{IIB}}_{t}=\mathrm{HB},$$

For driven only by norms, we set *Impact*_*HBonIIB*_ to 0 in Equation 1, making *IIB* only dependent on (equal to) *Norm*:

$${\mathrm{IIB}}_{t}={\text{Impact}}_{\text{HBonIIB}}\times \mathrm{HB}+\left(1-{\text{Impact}}_{\text{HBonIIB}}\right)\times {\text{Norm}}_{t},$$

$${\mathrm{IIB}}_{t}=0\times \mathrm{HB}+\left(1-0\right)\times {\text{Norm}}_{t},$$

$${\mathrm{IIB}}_{t}={\text{Norm}}_{t}.$$

For driven by health awareness and norms combined, we leave Equation 1 as it is, with *Impact*_*HBonIIB*_ as estimated from the cohort data using regression through the origin (Table 4), where *IIB* is dependent on both *HB* and *Norm*:

$${\mathrm{IIB}}_{t}={\text{Impact}}_{\text{HBonIIB}}\times \mathrm{HB}+\left(1-{\text{Impact}}_{\text{HBonIIB}}\right)\times {\text{Norm}}_{t}.$$

## 3 RESULTS

We divide the results in three sections corresponding to the results of the (1) optimization, (2) validation and (3) scenarios.

### 3.1 Optimization

Table 6 shows the optimal values for *Intent*_*PAB*_ and *Intent*_*EB*_ for each group, referring to the change that individuals make in eating behaviour and physical activity behaviour. These are expressed as a change in *PAL* and in *TDEI* per unit of *BMI* that their *BMI* differs from their *IIB* (their target weight loss/gain expressed by *Discrepancy*_*BMIandIIB*_). South‐Asian Surinamese women, for instance, decrease their energy intake by 234 kcal/day for each unit of *BMI* that they consider to be excessive, according to the cohort data in conjunction with the data‐generating assumptions (see Appendix S2.1 for the cost function values for each set). In Appendix S2.1, we also show that the SDMs are robust against small changes in these parameter values.

**TABLE 6:** Optimization results for each group

Sociocultural group	*Intent*_*EB*_ in (kcal/day)/(kg/m^2^)	*Intent*_*PAB*_ in 1/(kg/m^2^)
Dutch men	−295	0.056
Moroccan men	−301	0.056
South‐Asian Surinamese men	−283	0.055
Dutch women	−251	0.060
Moroccan women	−259	0.061
South‐Asian Surinamese women	−234	0.057

### 3.2 Validation

We obtain a validation score of 20/32 points (62.5% correct) for the set of SDMs. This implies that the set of equations that we use is capable of capturing the expected behaviour to a large extent. Table 7 shows for each behaviour whether it is exhibited by the respective SDMs.

**TABLE 7:** Behaviours exhibited by the SDMs.

	Validation statement	Operationalization: Behaviour exhibited by the SDMs?
1	There are no significant physiological differences between Dutch and South‐Asian Surinamese men regarding the effect of physical activity on weight loss.	Yes: In the male groups, the effect of *PAL* on *BMI* is of the same order of magnitude in the Dutch as the South‐Asian Surinamese group.
2	There are no significant physiological differences between Dutch and Moroccan men regarding the effect of physical activity on weight loss.	Yes: In the male groups, the effect of *PAL* on *BMI* is of the same order of magnitude in the Dutch as the Moroccan group.
3	There are no significant physiological differences between South‐Asian Surinamese and Moroccan men regarding the effect of physical activity on weight loss.	Yes: In the male groups, the effect of *PAL* on *BMI* is of the same order of magnitude in the South‐Asian Surinamese as the Moroccan group.
4	There are no significant physiological differences between Dutch and South‐Asian Surinamese women regarding the effect of physical activity on weight loss.	Yes: In the female groups, the effect of *PAL* on *BMI* is of the same order of magnitude in the Dutch as the South‐Asian Surinamese group.
5	There are no significant physiological differences between Dutch and Moroccan women regarding the effect of physical activity on weight loss.	Yes: In the female groups, the effect of *PAL* on *BMI* is of the same order of magnitude in the Dutch as the Moroccan group.
6	There are no significant physiological differences between South‐Asian Surinamese and Moroccan women regarding the effect of physical activity on weight loss.	Yes: In the female groups, the effect of *PAL* on *BMI* is of the same order of magnitude in the South‐Asian Surinamese as the Moroccan group.
7	There are no significant physiological differences between Dutch men and women regarding the effect of physical activity on weight loss.	Yes: In the Dutch group, the effect of *PAL* on *BMI* is of the same order of magnitude in the male as in the female group.
8	There are no significant physiological differences between South‐Asian Surinamese men and women regarding the effect of physical activity on weight loss.	Yes: In the South‐Asian Surinamese group, the effect of *PAL* on *BMI* is of the same order of magnitude in the male as in the female group.
9	There are no significant physiological differences between Moroccan men and women regarding the effect of physical activity on weight loss.	Yes: In the Moroccan group, the effect of *PAL* on *BMI* is of the same order of magnitude in the male as in the female group.
10	Norms tend to have a larger effect on Moroccan than on Dutch men, as Moroccan culture is regarded as being more collectivistic than Dutch culture.	Yes: In the male groups, (1—*Impact*_*HBonIIB*_), representing the impact of *Norm* on *IIB*, is higher in the Moroccan than in the Dutch group.
11	Norms tend to have a larger effect on South‐Asian Surinamese than on Dutch men, as South‐Asian Surinamese culture is regarded as being more collectivistic than Dutch culture.	No: In the male groups, (1—*Impact*_*HBonIIB*_), representing the impact of *Norm* on *IIB*, is not higher in the South‐Asian Surinamese than in the Dutch group.
12	Norms tend to have a larger effect on Moroccan than on South‐Asian Surinamese men, as Moroccan culture is regarded as being more collectivistic than South‐Asian Surinamese culture.	Yes: In the male groups, (1—*Impact*_*HBonIIB*_), representing the impact of *Norm* on *IIB*, is higher in the Moroccan than in the South‐Asian Surinamese group.
13	Norms tend to have a larger effect on Moroccan than on Dutch women, as Moroccan culture is regarded as being more collectivistic than Dutch culture.	No: In the female groups, (1—*Impact*_*HBonIIB*_), representing the impact of *Norm* on *IIB*, is not higher in the Moroccan than in the Dutch group.
14	Norms tend to have a larger effect on South‐Asian Surinamese than on Dutch women, as South‐Asian Surinamese culture is regarded as being more collectivistic than Dutch culture.	No: In the female groups, (1—*Impact*_*HBonIIB*_), representing the impact of *Norm* on *IIB*, is not higher in the South‐Asian Surinamese than in the Dutch group.
15	Norms tend to have a larger effect on Moroccan than on South‐Asian Surinamese women, as Moroccan culture is regarded as being more collectivistic than South‐Asian Surinamese culture.	Yes: In the female groups, (1—*Impact*_*HBonIIB*_), representing the impact of *Norm* on *IIB*, is higher in the Moroccan than in the South‐Asian Surinamese group.
16	Dutch women tend to be more prone to be influenced by norms than Dutch men.	No: In the Dutch group, (1—*Impact*_*HBonIIB*_), representing the impact of *Norm* on *IIB*, is not higher in the female than in the male group.
17	South‐Asian Surinamese women tend to be more prone to be influenced by norms than South‐Asian Surinamese men.	No: In the south‐Asian Surinamese group, (1—*Impact*_*HBonIIB*_), representing the impact of *Norm* on *IIB*, is not higher in the female than in male group.
18	Moroccan women tend to be more prone to be influenced by norms than Moroccan men.	No: In the Moroccan group, (1—*Impact*_*HBonIIB*_), representing the impact of *Norm* on *IIB*, is not higher in the female than in the male group.
19	The intent to change physical activity behaviour tends to be greater in Dutch than in Moroccan men, as physical activity is more embedded in Dutch than in Moroccan culture.	No: In the male groups, *Intent*_*PAB*_ is not higher in the Dutch than in the Moroccan group.
20	The intent to change physical activity behaviour tends to be greater in Dutch than in South‐Asian Surinamese men, as physical activity is more embedded in Dutch than in South‐Asian Surinamese culture.	Yes: In the male groups, *Intent*_*PAB*_ is higher in the Dutch than in the South‐Asian Surinamese group.
21	The intent to change physical activity behaviour tends to be greater in Dutch than in Moroccan women, as physical activity is more embedded in Dutch than in Moroccan culture.	No: In the female groups, *Intent*_*PAB*_ is not higher in the Dutch than in the Moroccan group.
22	The intent to change physical activity behaviour tends to be greater in Dutch than in South‐Asian Surinamese women, as physical activity is more embedded in Dutch than in South‐Asian Surinamese culture.	Yes: In the female groups, *Intent*_*PAB*_ is higher in the Dutch than in the South‐Asian Surinamese group.
23	The intent to change physical activity behaviour tends to be greater in Dutch women than in Dutch men.	Yes: In the Dutch group, *Intent*_*PAB*_ is higher in the female than in the male group.
24	The intent to change physical activity behaviour tends to be greater in South‐Asian Surinamese men than in South‐Asian Surinamese women.	No: In the South‐Asian Surinamese group, *Intent*_*PAB*_ is not higher in the male than in the female group.
25	The intent to change physical activity behaviour tends to be greater in Moroccan men than in Moroccan women.	No: In the Moroccan group, *Intent*_*PAB*_ is not higher in the male than in the female group.
26	The intent to change eating behaviour tends to be greater in Dutch than in Moroccan men, as eating behaviour is more important in Moroccan than in Dutch culture.	No: In the male groups, *Intent*_*EB*_ is not higher in the Dutch than in the Moroccan group.
27	The intent to change eating behaviour tends to be greater in Dutch than in South‐Asian Surinamese men, as eating behaviour is more important in South‐Asian Surinamese than in Dutch culture.	Yes: In the male groups, *Intent*_*EB*_ is higher in the Dutch than in the South‐Asian Surinamese group.
28	The intent to change eating behaviour tends to be greater in Dutch than in Moroccan men, as eating behaviour is more important in Moroccan than in Dutch culture.	No: In the female groups, *Intent*_*EB*_ is not higher in the Dutch than in the Moroccan group.
29	The intent to change eating behaviour tends to be greater in Dutch than in South‐Asian Surinamese women, as eating behaviour is more important in South‐Asian Surinamese than in Dutch culture.	Yes: In the female groups, *Intent*_*EB*_ is higher in the Dutch than in the South‐Asian Surinamese group.
30	The intent to change eating behaviour tends to be greater in Dutch women than in Dutch men.	Yes: In the Dutch group, *Intent*_*EB*_ is higher in the female than in the male group.
31	The intent to change eating behaviour tends to be greater in Moroccan women than in Moroccan men.	Yes: In the Moroccan group, *Intent*_*EB*_ is higher in the female than in the male group.
32	The intent to change eating behaviour tends to be greater in South‐Asian Surinamese women than in South‐Asian Surinamese men.	Yes: In the South‐Asian Surinamese group, *Intent*_*EB*_ is higher in the female than in the male group.

*Note*: Each validation statement is supported by 1–8 references, as we show in Appendix S1.5. In the right column, we show whether the behaviour is exhibited by the respective pair of SDMs based on the computational operationalization of the validation statement (see Appendix S1.5).

Abbreviation: SDM, system dynamics model.

### 3.3 Scenarios

**FIGURE 4:**
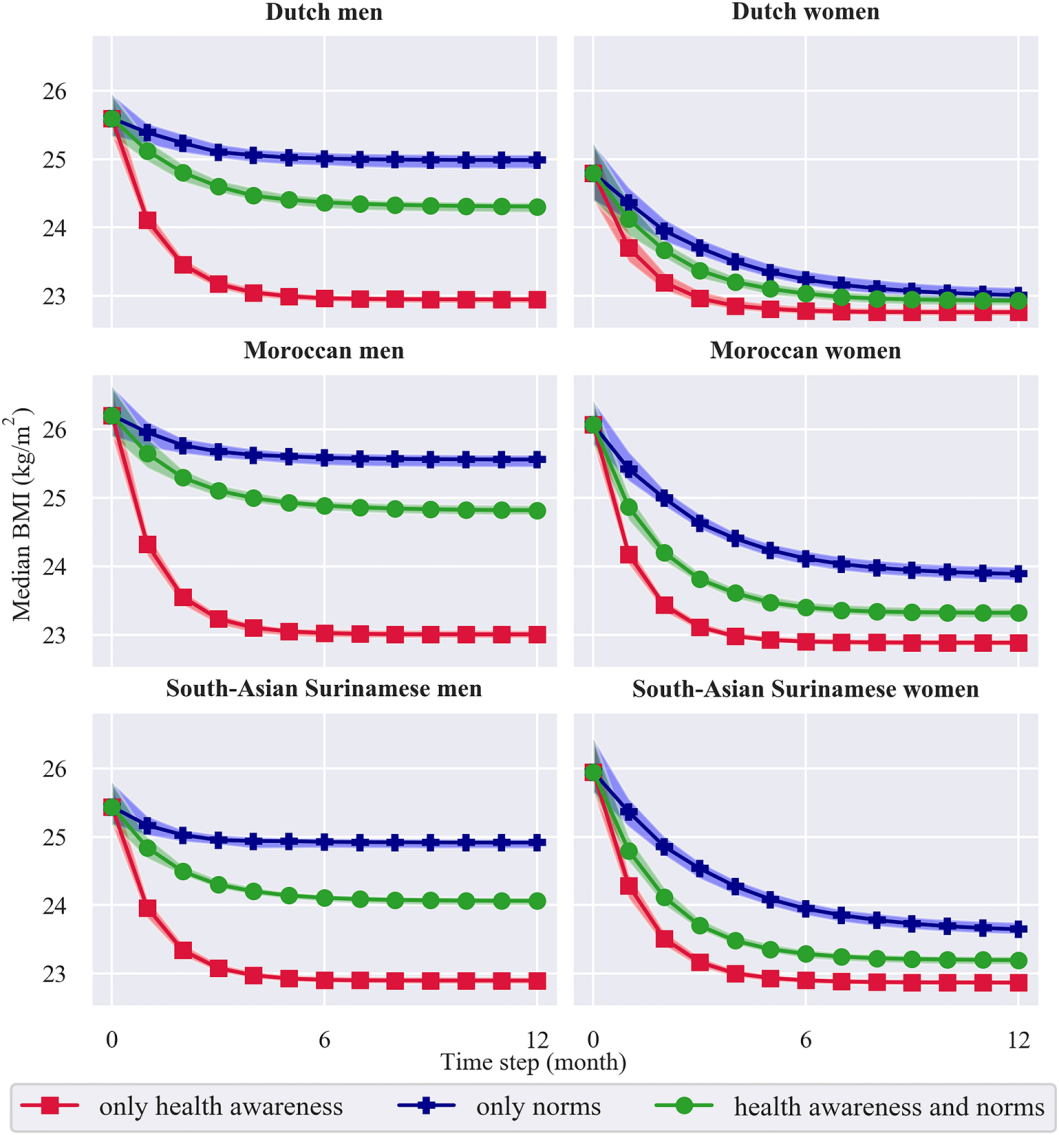
Change in group‐level body mass index (BMI) over time for three scenarios: ‘what if' weight‐related behaviour were driven (1) only by health awareness (red), (2) only by norms (blue) and (3) by health awareness and norms combined (green). The 95% confidence interval (details in Appendix S2.2) corresponding to each of these scenarios is shaded in red, blue and green, respectively

For all groups (Figure 4), we see that if weight‐related behaviour were driven by a combination of health awareness and norms (green), then group‐level median BMI would be lower than if driven only by norms (blue) and higher than if driven only by health awareness (red). If driven only by health awareness (red), there would only be minor differences between the male and female groups: median BMI would drop 11% (3 BMI points) among groups of both genders, which is trivial given the definition of this scenario.

For the other scenarios, that is, if driven only by norms (blue) and by health awareness and norms combined (green), we see different emergent behaviours between the male versus female groups, while they demonstrate only slight differences between groups of the same gender. These small quantitative differences (given in Appendix S2.2) are not as reliable as the large qualitative differences that our simulations show, given the cross‐sectional data that we use. We therefore mostly focus on the qualitative differences, that is, those between genders.

If driven only by norms (blue), there would be an average reduction in median BMI of 5% (1 point). However, we see that among the male groups, median BMI would only decrease by 2% (1 point), whereas among the female groups, it would reduce by 8% (2 points). This pattern is observed in all three ethnic groups.

If driven by health awareness and norms combined (green), median BMI would drop 7% (2 points) on average. Again, it would decrease less in the male groups, with only 5% (1 point). In the female groups, the decrease would be larger: 10% (2 points).

The male groups thus demonstrated an over two times larger drop if driven by health awareness than if driven by health awareness and norms combined. In contrast, their female counterparts showed a more equal drop in both these scenarios. When driven only by norms, median BMI in all groups would decrease to a lesser extent. In the female groups, however, being driven only by norms leads to a similar decrease in median BMI as being driven only by health awareness and being driven by health awareness and norms combined. In the male groups, the decrease in median BMI is different for all scenarios.

## 4 DISCUSSION

We constructed SDMs of the system of social norms regarding body weight perception and obesity prevalence, which we used to compare the effect of health awareness versus norms on group‐level BMI. These SDMs were developed to explicitly describe the causal mechanism underlying this system and to get insights into the long‐term consequences of this mechanism at the group level. Our results show that group‐level BMI shifts to a lesser extent in all groups if norms have an influence on weight‐related behaviour, confirming that norms are counteracting health awareness in shaping this behaviour. Particularly in groups where overweight is the norm, that is, all male groups, the norm holds group‐level BMI close to overweight despite of health awareness.

These results should be seen in light of a number of methodological considerations. We opted for SDMs as they allow us to assume that within a group all individuals compare themselves to that group equivalently, that is, their interactions are homogeneous. We did not have the data necessary to model possible heterogeneity in behaviour nor in social connectivity, as would be required to make, for example, an agent‐based modelling approach^40^—with diverse interactions of unique agents with others and with their environment—valuable. We also assumed that there is no interaction between the sociocultural groups with respect to this system, implying that individuals are most likely to compare themselves to others from their own sociocultural group. Although we cannot rule out that there is interaction affecting this system, the similarity hypothesis that we based our assumption on is well‐established.^3^ In our SDMs, individuals compare themselves to their entire sociocultural group, whereas this comparison group might actually be more confined. Stratifying by other characteristics that also affect body weight perception and the influence of norms in addition to gender and ethnicity, for example, age,^41^ would result in smaller comparison groups. Zooming in on these subgroups could reveal additional emergent behaviours, which we did not detect in this study as we focused on the current groups' average dynamics.

In addition, we assumed that median BMI and sociocultural ideal BMI exert an equal influence on the norm, as the most conservative ratio, which might not be accurate. We also assumed that individuals adjust their weight‐related behaviour to narrow the gap between their actual and ideal BMI and that their behaviour is driven by health awareness and norms. Our assumption implies that these are the only determinants of weight change, which is a simplification of reality. We deliberately kept the scope of the SDMs limited, omitting other drivers of weight‐related behaviour. This is related to the proof‐of‐concept function of this study, where we intended to demonstrate the methods required to design functioning SDMs with the aim to study feedback loops relevant to the field of public health. The SDMs therefore include only those factors that we deem necessary and sufficient to describe this system as the aim is to understand the implications of its feedback loops on the long term rather than to propose interventions or make predictions. In reality, broader environmental variables as well as individual characteristics such as education and income could affect whether individuals are willing and able to adjust their behaviour. Given that the aim of our SDMs was to unravel a specific causal mechanism and its long‐term effects at the group level, we did not deem it essential to take individual physiological factors or genetic predispositions into account. Nevertheless, we acknowledge that these factors have a role to play in weight loss and weight gain at the individual level. We argue, however, that norms and their impact on weight‐related behaviour are crucial factors to study in light of the obesity epidemic as changes in physiology or genetics at the group level are unable to exclusively explain the trend in obesity rates. All other variables and characteristics being equal, our SDMs show the impact of norms in groups that differ regarding body weight perception, group‐level norms and the strengths of the causal links between them. The SDMs thus do not constitute a description of reality but serve to explore ‘what if' scenarios in a restricted, specific context. Our SDMs may serve as seed models for iterative extensions to include additional variables, which would make weight‐related behaviour dependent on more determinants, possibly introducing more divergence in emergent behaviour. We however considered it important to understand the impact of norms in isolation before compounding additional causal mechanisms into the same SDMs.

As the data were cross‐sectional, we used expert‐informed data‐generating assumptions to optimize the values for the parameters for intent to change physical activity and eating behaviour, respectively. This means that in each group, we based our simulations on the same hypothesized fundamental behaviours, as supported by literature, with respect to weight gain/loss, for example, concerning physiological constraints (rate of weight change). These fundamental behaviours however do not markedly influence the differences we observe between the groups over time. These differences are primarily based on the initial characteristics and the fitted parameters, which differ per group, in conjunction with the equations.

Lastly, structural validation is ideally followed by behaviour validation. Behaviour validation, however, requires the use of longitudinal data, that is, data that enable us to validate the behaviour patterns over time. Still, the sequence of the formal model validation for SDMs stages implies that we validated our SDMs according to the gold standard in the case that longitudinal data are unavailable. In practice, behaviour validation is often used without going through structural validation. Given the inherent importance of the latter in system dynamics modelling in particular, that is, ‘it is often said that a system dynamics model must generate “the right output for the right reasons”',^39^ our efforts concerning this type of validity can be seen as an asset of our research. The manner in which we addressed the structural validation of our SDMs—for example, by supporting the expert‐informed CLD with literature, using established equations, maintaining dimensional consistency and employing validation statements—serves to establish that the assumptions we made currently hold. Nevertheless, this does not rule out the possibility that new empirical evidence might call for readjustments of these assumptions.

Our results can be placed in the context of the current debate on addressing group‐level determinants in policy aiming to reduce obesity prevalence. In public health, it seems widely accepted that solutions for the increasing rates of overweight and obesity cannot solely be found in individual‐level actions such as health education. An additional focus on group‐level determinants such as the food environment or norms regarding body weight seems imperative. Although policies often start with a commitment to deal with group‐level determinants, they frequently end up with the implementation of individualized interventions aimed at changing individual weight‐related behaviour.^42^, ^43^ A failure to tackle group‐level determinants may be because—although there is a large body of knowledge on the way these determinants shape public health problems—the knowledge base regarding the impact of interventions addressing these determinants is limited.

Currently, the evaluation of interventions aimed at higher level determinants is largely dependent on ‘natural experiments', an approach that has proven to be extremely complex and heavily dependent on the very few actual policies that lend themselves for impact evaluation.^44^ The use of complexity science, more specifically systems dynamic modelling, applied to relevant processes at the group level, is likely to speed up knowledge production in this field. Our study is an attempt to explore the value of this approach, specifically for the issue of tackling overweight and obesity. The results of this study should not be interpreted as predictions that can directly be applied to policy but rather as ‘what if' scenarios that attempt to clarify a causal mechanism. The SDMs enable us to untangle this mechanism by exhibiting patterns that we could not have predicted by only considering group‐level characteristics. For instance, this way of considering the individual values for sociocultural ideal BMI—which during the simulations remain initialized by every individual's own value for this variable as derived from the cohort data—presents us with a group‐level pattern that we could not have predicted otherwise. This pattern that we observe at the group level can be interpreted as an emergent behaviour of the system. What lessons can be drawn from our study regarding the underlying causal mechanism we modelled?

Firstly, this study adds understanding to how group‐level processes, in this case in relation to norms, work. Specifically, previous research has shown that obesity spreads through social ties.^45^ Our work explores a potential causal mechanism for this process by specifying that individual body weight perception is affected by what is normal while also affecting what is normal via its impact on obesity prevalence, inducing feedback loops that may over time contribute to group‐level obesity. We investigated this hypothesized causal mechanism by operationalizing it using system dynamics modelling, where all causal links are supported by literature and implemented mathematically in an intuitive, explainable manner. This is contrary to the majority of other quantitative epidemiological methods, which are based on statistical models attempting to discover correlations—without explicitly studying causal mechanisms. Our approach enables us to simulate each group's emergent behaviour as a function of the hypothesized causal mechanisms. The results of this study confirm that the hypothesized feedback loops can be an explanation for group‐level obesity, where group‐level BMI is reinforced by individual body weight perception via the norm.

In this study, we also show that if norms were the only determinant of weight change, men would not lose weight, whereas women would. This implies that norms are counteracting health awareness less strongly in women, suggesting that they must be subject to additional drivers of obesity. The major driver of this divergence in behaviour is the norm, which is closer to the healthy weight range in women. This is in line with previous research showing that women are generally more engaged in health and weight‐related behaviour.^46^, ^47^ Previous research has also shown ‘that appearance norms encountered by women in daily life are more rigid, homogeneous and pervasive than those for men, and that more messages implying the attainability of the ideal appearance are directed at women'.^48^ For women, having this pressure to conform to the sociocultural ideal BMI might add to the discrepancy between the results of the scenarios and reality. That is, despite of being aware of the sociocultural ideal BMI and the sociocultural ideal BMI being healthy, which under our assumptions should lead to weight loss in women, we see in reality that, at the group level, women are not losing weight. In this regard, it has for instance been shown that weight stigma increased the inclination to avoid exercise, independent of BMI and body satisfaction, among college‐aged females.^49^

In addition, under our assumptions, the cohort data underlying our SDMs suggest that the relative impact of norms on individual ideal BMI is larger among men. This is of interest, as we usually consider the effect of norms on weight change to be indicative of their strength. That is, norms towards body weight are generally regarded as being stronger in women, as women more likely to want to adjust their weight based on norms.^50^ Our results however show that norms are stronger in men. This suggests that even though for men norms do not contribute to weight loss, they do establish the reinforcing feedback loops of group‐level obesity. As the cohort data also show that the norm is approximately equal to group‐level BMI in men, this might indicate that what is normal may be more important than what is ideal in determining what body weight men strive for. This is supported by findings from previous research showing that men are less likely to recognize their own overweight status and are more satisfied with their body weight than women.^51^, ^52^

Secondly, this study adds evidence that efforts to address the obesity epidemic need to consider norms, as an example of group‐level processes. Our results indicate that in populations where overweight is the norm, the potential impact of policies is greater if they address these norms, as compared with policies that use individual‐level approaches only. Here, addressing the norm does not equal enforcing stricter weight ideals, as the norm is dependent on both median BMI *and* sociocultural ideal BMI—where the latter refers to weight ideals. The norm is thus influenced by median BMI, that is, what you see around you. From a policy perspective, this might mean that making individuals aware of their obesity status is not the road forward, but taking measures at the group level to reduce the obesity prevalence is. A commitment to deal with the norm can thus be independent of sociocultural ideal BMI, by attending to median BMI at the group level.

Studying ‘what if' scenarios using computational modelling approaches allows us to test policy strategies under the exact same conditions. Although the SDMs do not include potential solutions for influencing norms, they show that we should do justice to influences on both the individual as well as the group level. Our results suggest that norms limit the effectiveness of interventions targeting individual weight‐related behaviour, especially in men. A restricted focus on individual weight‐related behaviour, which places the responsibility for body weight on the individual in a context where overweight is the norm, might even have adverse effects.^53^ The results of this study indicate that shifting the focus to group‐level interventions aiming to change the norm can contribute to the prevention of group‐level obesity. A starting point for this can be considering how the effects of policy strategies may diverge among groups based on the prevailing norm towards body weight. Given that in our study population overweight prevalence is high in both men and women, the results of our scenarios imply that failing to consider the norm and its effect over time might result in a similar policy strategy for both genders, which would be misguided. Our SDMs can be instrumental in considering the norms of groups that, besides gender and ethnicity, differ with respect to characteristics such as socio‐economic status. This can be explored in future research using these SDMs and would allow us to differentiate policy based on the group‐level norm towards body weight, in addition to obesity prevalence.

## 5 CONCLUSIONS

We showed that norms regarding body weight perception withhold individuals from losing as much weight as they could if driven by health awareness alone. When overweight is the norm, that is, in all male groups, the norm holds group‐level BMI close to overweight despite of health awareness. Our results thus suggest that norms limit the effectiveness of interventions targeting individual weight‐related behaviour, especially in men. Since norms are counteracting health awareness less strongly in the female groups, there must be additional drivers of obesity in women.

## CONFLICT OF INTEREST

No conflict of interest was declared.

## AUTHOR CONTRIBUTIONS

LC, MN and KS conceptualized the study. PD, LC, RQ, NM and PMAS were responsible for the computational modelling component of the study. All authors were involved in the interpretation of the results and in the writing of the manuscript. All authors approved the final version.

## Supporting information


**Figure S1.** Visual representation of the calibration between perceived BMI as indicated by the body image scale and mean measured BMI for each group. The solid line represents the linear regression line between the dependent variable y = perceived BMI as indicated by the body image scale, and the independent variable x = mean measured BMI. Accordingly, the dots represent the mean measured BMI corresponding to each of the nine images from the body image scale.
**Figure S2.** Regions corresponding to different behaviours with respect to *HB* and *Norm*. Each blue dot represents 1–370 individuals that have that particular combination of distance *Norm* and *HB* and distance *Norm* and *IIB*. The distance between *Norm* and *HB* (x‐axis) can only have a limited amount of values. This is because *Norm* depends on *MedBMI* and *SCIB*, where *SCIB* only has nine possible values (as it is estimated based on the body image scale), and because *HB* is the same for all individuals (22.5 kg/m^2^). The distance between *Norm* and *IIB* (y‐axis) can only have a fairly limited amount of values because there are also only nine possible values for *IIB* (as it is estimated based on the body image scale).
**Figure S3.** Energy landscape and behaviour of attractor for stock *s*. The green dot represents the attractor point (*s*_*optimal*_) which is at stock value *s* = 0 and the red dots represent the individuals, whose motion is towards the attractor point as indicated by the red arrows.
**Figure S4.** Illustration of local linearity.^16^
**Figure S5.** The age distributions of the cohort data of the HELIUS study, the population of the Netherlands, and the population of Amsterdam.
**Figure S6.** Test 1 of sensitivity analysis of I*ntent*_*EB*_ and I*ntent*_*PAB*_ on *MedBMI* for South‐Asian Surinamese men. We vary *Intent*_*EB*_ whilst keeping *Intent*_*PAB*_ fixed at its optimal value.
**Figure S7.** Test 1 of sensitivity analysis of *Intent*_*EB*_ and *Intent*_*PAB*_ on *MedBMI* for South‐Asian Surinamese women. We vary *Intent*_*EB*_ whilst keeping *Intent*_*PAB*_ fixed at its optimal value.
**Figure S8.** Test 2 of sensitivity analysis of *Intent*_*EB*_ and *Intent*_*PAB*_ on *MedBMI* for South‐Asian Surinamese men. We vary *Intent*_*PAB*_ whilst keeping *Intent*_*EB*_ fixed at its optimal value.
**Figure S9.** Test 1 of sensitivity analysis of *Intent*_*EB*_ and *Intent*_*PAB*_ on *MedBMI* for South‐Asian Surinamese women. We vary *Intent*_*EB*_ whilst keeping *Intent*_*PAB*_ fixed at its optimal value.
**Table S1.** Results of Kruskal Wallis H tests to compare mean ranks between ethnic groups of the same gender (male groups).
**Table S2.** Results of Kruskal Wallis H tests to compare mean ranks between ethnic groups of the same gender (female groups).
**Table S3.** Results of two‐sample Kolmogorov–Smirnov tests to compare distributions between male and female groups of the same ethnicity.
**Table S4.** The fitted α value for each group.
**Table S5.** Validation statements.
**Table S6.** Number of validation statements (out of 32) satisfied by the SDMs according to the original age distribution of the cohort data of the HELIUS study, reflecting the age distribution of the population of the Netherlands, and reflecting the age distribution of the population of Amsterdam.
**Table S7.** Behaviours exhibited by the SDMs according to the actual cohort data of the HELIUS study, reflecting the age distribution of the population of the Netherlands, and reflecting the age distribution of the population of Amsterdam.
**Table S8.** Optimization results including value for the cost function corresponding to the set of optimized parameters.
**Table S9.** Sensitivity analysis of *Impact*_*HBonIIB*_, showing the percentage change in stable MedBMI for a 50% increase and decrease in the value for *Impact*_*HBonIIB*_.
