# Supplementary material for: Social norms and obesity prevalence: From cohort to system dynamics models
Source: Obes Rev. 2020 May 13;21(9):e13044. doi: 10.1111/obr.13044 (PMC7507199; doi:10.1111/obr.13044)
Supplement: Supplementary file 1 — Figure S1. Visual representation of the calibration between perceived BMI as indicated by the body image scale and mean measured BMI for each group. The solid line represents the linear regression line between the dependent variable y = perceived BMI as indicated by the body image scale, and the independent variable x = mean measured BMI. Accordingly, the dots represent the mean measured BMI corresponding to each of the nine images from the body image scale. Figure S2. Regions corresponding to different behaviours with respect to HB and Norm. Each blue dot represents 1–370 individuals that have that particular combination of distance Norm and HB and distance Norm and IIB. The distance between Norm and HB (x‐axis) can only have a limited amount of values. This is because Norm depends on MedBMI and SCIB, where SCIB only has nine possible values (as it is estimated based on the body image scale), and because HB is the same for all individuals (22.5 kg/m2). The distance between Norm and IIB (y‐axis) can only have a fairly limited amount of values because there are also only nine possible values for IIB (as it is estimated based on the body image scale). Figure S3. Energy landscape and behaviour of attractor for stock s. The green dot represents the attractor point (s optimal) which is at stock value s = 0 and the red dots represent the individuals, whose motion is towards the attractor point as indicated by the red arrows. Figure S4. Illustration of local linearity.16 Figure S5. The age distributions of the cohort data of the HELIUS study, the population of the Netherlands, and the population of Amsterdam. Figure S6. Test 1 of sensitivity analysis of Intent EB and Intent PAB on MedBMI for South‐Asian Surinamese men. We vary Intent EB whilst keeping Intent PAB fixed at its optimal value. Figure S7. Test 1 of sensitivity analysis of Intent EB and Intent PAB on MedBMI for South‐Asian Surinamese women. We vary Intent EB whilst keeping Intent PAB fixed at its optimal value. [file OBR-21-e13044-s001.docx]

**Appendix**

**Social norms and obesity prevalence: from cohort to system dynamics models**

Loes Crielaard MSc^†1,2^, Pritha Dutta M.E.^†3,4^, Rick Quax PhD^2,5^, Mary Nicolaou PhD^1,2^, Nadège Merabet PhD^1,2^, Prof Karien Stronks PhD^1,2^, Prof Peter M. A. Sloot PhD^2,3,5^

^†^Contributed equally

^1^ Department of Public Health, Amsterdam UMC, University of Amsterdam, Amsterdam Public Health Research Institute, Meibergdreef 9, Amsterdam, The Netherlands

^2^ Institute for Advanced Study, University of Amsterdam, Amsterdam 1012 GC, The Netherlands

^3^ Complexity Institute, Nanyang Technological University, Singapore 637335, Singapore

^4^ Interdisciplinary Graduate Programme, Nanyang Technological University, Singapore 637335, Singapore

^5^ Computational Science Lab, University of Amsterdam, Amsterdam 1098 XH, The Netherlands

Correspondence to: Loes Crielaard MSc, Department of Public Health, Amsterdam UMC, University of Amsterdam, Amsterdam Public Health Research Institute, Meibergdreef 9, Amsterdam, The Netherlands, **l.crielaard@amsterdamumc.nl**, **+31205661562**

**Table of contents**

[1. Methods 3](#_Toc33624519)

[1.1 Study population 3](#_Toc33624520)

[o Statistical analysis confirming diversity in body weight perception 3](#_Toc33624521)

[o Linear regression confirming exclusion of age from causal loop diagram 4](#_Toc33624522)

[o Data collection 4](#_Toc33624523)

[1.2 Body weight perception 4](#_Toc33624524)

[1.3 Variables and equations 5](#_Toc33624525)

[o Anthropometric and demographic variables 5](#_Toc33624526)

[o Energy balance variables 5](#_Toc33624527)

[o *Impact_HBonIIB_* as unique determinant of an individual’s behaviour with respect to *HB* and *Norm* 6](#_Toc33624528)

[1.4 From cross-sectional data to temporal dynamics 8](#_Toc33624529)

[o Summary of previously proposed methods to generate pseudo-longitudinal data from cross-sectional data 8](#_Toc33624530)

[o Relation to energy landscape, approximation of local linearity, and data-generating assumptions 8](#_Toc33624531)

[o Cost function 12](#_Toc33624532)

[1.5 Validation statements and weighting cohort data of the HELIUS study 13](#_Toc33624533)

[o Validation statements 13](#_Toc33624534)

[o Weighting cohort data of the HELIUS study 19](#_Toc33624535)

[2. Results 22](#_Toc33624536)

[2.1 Optimisation 22](#_Toc33624537)

[o Optimisation results 22](#_Toc33624538)

[o Sensitivity analysis of *Intent_EB_* and *Intent_PAB_* 22](#_Toc33624539)

[2.2 Scenarios 25](#_Toc33624540)

[o 95% confidence interval 25](#_Toc33624541)

[o Differences between socio-cultural groups in change in group-level BMI over time for scenarios 25](#_Toc33624542)

[o Sensitivity analysis of *Impact_HBonIIB_* 25](#_Toc33624543)

[3. References 26](#_Toc33624544)

# Methods

## Study population

### Statistical analysis confirming diversity in body weight perception

Differences in body weight perception between the socio-cultural groups are verified with statistical analyses using Kruskal Wallis H tests (an analysis of variance (ANOVA) test could not be used as parametric assumptions were violated), followed by Dunn’s post-hoc tests, as well as two-sample Kolmogorov-Smirnov tests. We want to compare distributions between ethnic groups of the same gender and between male and female groups of the same ethnicity, as it does not make sense to compare groups of both different ethnicity and different gender with respect to body weight perception.

Preferably, we would like to test for any differences among the groups’ distributions, not only those based on their mean ranks (as we can detect with the Kruskal Wallis H tests). This is because we consider the distributions as energy landscapes: we want to know whether the landscapes differ among the groups (see ***Appendix 1.4*** for relation to energy landscape). The two-sample Kolmogorov-Smirnov test can be used for this. However, this test can only be used for comparing two groups, whereas we also want to compare distributions between ethnic groups of the same gender (three groups). We therefore use Kruskal Wallis H tests to compare ethnic groups of the same gender (table S1 and table S2) and two-sample Kolmogorov-Smirnov tests to compare male and female groups of the same ethnicity (table S3) (both *p*<0.05).

After the Kruskall Wallis H tests, Dunn’s post-hoc tests (*p*<0.05) are performed to investigate the pairwise differences between ethnic groups of the same gender. This is to pinpoint which specific mean ranks differ significantly from the others.

***Table S1:* Results of Kruskal Wallis H tests to compare mean ranks between ethnic groups of the same gender (male groups).**

|  | Dutch men  (*n*=753) | Moroccan men  (*n*=774) | South-Asian Surinamese men  (*n*=839) |  | | |
| --- | --- | --- | --- | --- | --- | --- |
|  | median (kg/m^2^) | median (kg/m^2^) | median (kg/m^2^) | df | χ^2^ | *p* |
| Perceived BMI | 25.37 | 25.95 | 25.32 | 2 | 84.028 | <0.01 |
| Individual ideal BMI (*IIB*) | 25.37 | 25.95 | 25.32 | 2 | 468.476 | <0.01 |
| Socio-cultural ideal BMI (*SCIB*) | 25.37 | 25.95 | 25.32 | 2 | 414.918 | <0.01 |

***Table S2:* Results of Kruskal Wallis H tests to compare mean ranks between ethnic groups of the same gender (female groups).**

|  | Dutch women  (*n*=848) | Moroccan women  (*n*=1,086) | South-Asian Surinamese women  (*n*=999) |  | | |
| --- | --- | --- | --- | --- | --- | --- |
|  | median (kg/m^2^) | median (kg/m^2^) | median (kg/m^2^) | df | χ^2^ | *p* |
| Perceived BMI | 25.02 | 25.73 | 25.06 | 2 | 111.707 | <0.01 |
| Individual ideal BMI (*IIB*) | 22.18 | 22.75 | 22.47 | 2 | 312.060 | <0.01 |
| Socio-cultural ideal BMI (*SCIB*) | 22.18 | 22.75 | 22.47 | 2 | 322.390 | <0.01 |

***Table S3:* Results of two-sample Kolmogorov-Smirnov tests to compare distributions between male and female groups of the same ethnicity.**

|  | Men and women (pooled) | | Dutch men and women | | Moroccan men and women | | South-Asian Surinamese men and women | |
| --- | --- | --- | --- | --- | --- | --- | --- | --- |
|  | *D*-statistic | *p* | *D*-statistic | *p* | *D*-statistic | *p* | *D*-statistic | *p* |
| Perceived BMI | 0.194 | <0.01 | 0.348 | <0.01 | 0.273 | <0.01 | 0.207 | <0.01 |
| Individual ideal BMI (*IIB*) | 0.589 | <0.01 | 0.612 | <0.01 | 0.615 | <0.01 | 0.542 | <0.01 |
| Socio-cultural ideal BMI (*SCIB*) | 0.621 | <0.01 | 0.664 | <0.01 | 0.616 | <0.01 | 0.590 | <0.01 |

These tests show that the mean ranks and distributions of the characteristics regarding body weight perception – perceived BMI, individual ideal BMI (*IIB*), and socio-cultural ideal BMI (S*CIB*) at *t*=0 (see subsection “**5. Variables and equations**” in the main manuscript) – are significantly different among the six groups. At least one pairwise difference is found in each Kruskal Wallis H test. Dunn’s post hoc tests show that for perceived BMI, *IIB*, and *SCIB*, all pairwise comparisons are significant among both male and female groups.

Based on these statistical analyses we conclude that there is significant diversity in body weight perception among Dutch, Moroccan, and South-Asian Surinamese men and women.

### Linear regression confirming exclusion of age from causal loop diagram

A causal link between age and individual ideal BMI was not included in the causal loop diagram (CLD). Still, during a feedback session on the preliminary structure of the system dynamics models (SDMs) at a later stage, Anita Hardon (anthropology) comments that age might have an effect on individual ideal BMI. Accordingly, she suggests that we should possibly either stratify by age in addition to by ethnicity and gender or incorporate a causal link between age and individual ideal BMI. We however do not find a relationship between these elements when investigating the link between age and individual ideal BMI in the cohort data and therefore decide to disregard this effect in this study.

### Data collection

The HEalthy Life in an Urban Setting (HELIUS) study is a prospective, multi-ethnic cohort study based in Amsterdam.^1^ The data obtained for this cohort that we use include characteristics concerning sex (which we use as a proxy for gender), age, ethnicity, education, BMI, and body weight perception for each individual. These data were collected through questionnaires/interviews, except for BMI which was obtained using a standardised methodology during a physical examination.^1,2^ Individuals with missing values for any of these characteristics are excluded. Ethnicity was based on an individual’s country of birth and those of his/her parents,^1,2^ where an individual was regarded of non-Dutch ethnic origin when they were born abroad with one or two parent(s) born abroad or born in the Netherlands with two parents born abroad.^1,2^ BMI was calculated as ${weight}/{{height}^{2}}$,^3^ averaged over two measurements taken while individuals were barefoot and lightly clothed.^1,2^ Body weight perception was self-reported^1,2^ (see subsection “**3. Body weight perception**” in the main manuscript for details). Education – classified into four groups: “no schooling or elementary schooling only”, “lower vocational schooling or lower secondary schooling”, “intermediate vocational schooling or intermediate/higher secondary schooling”, and “higher vocational schooling or university” – was self-reported and determined according to the highest qualification that was gained either in the Netherlands or the country of origin.^1,2^

## Body weight perception

For this cohort, perceived BMI was determined via questionnaires, asking each individual to indicate which image they most looked like on a randomly ordered version of the body image scale developed by Pulvers et al.^4^ (figure 2 in the main manuscript). For all groups we map each image to a corresponding measured BMI, where each image is represented by the average BMI for all individuals that selected that image as their perceived BMI (table 1 in the main manuscript). A visual representation of the calibration between perceived BMI as indicated by the body image scale and average measured BMI for each group is given in figure S1.


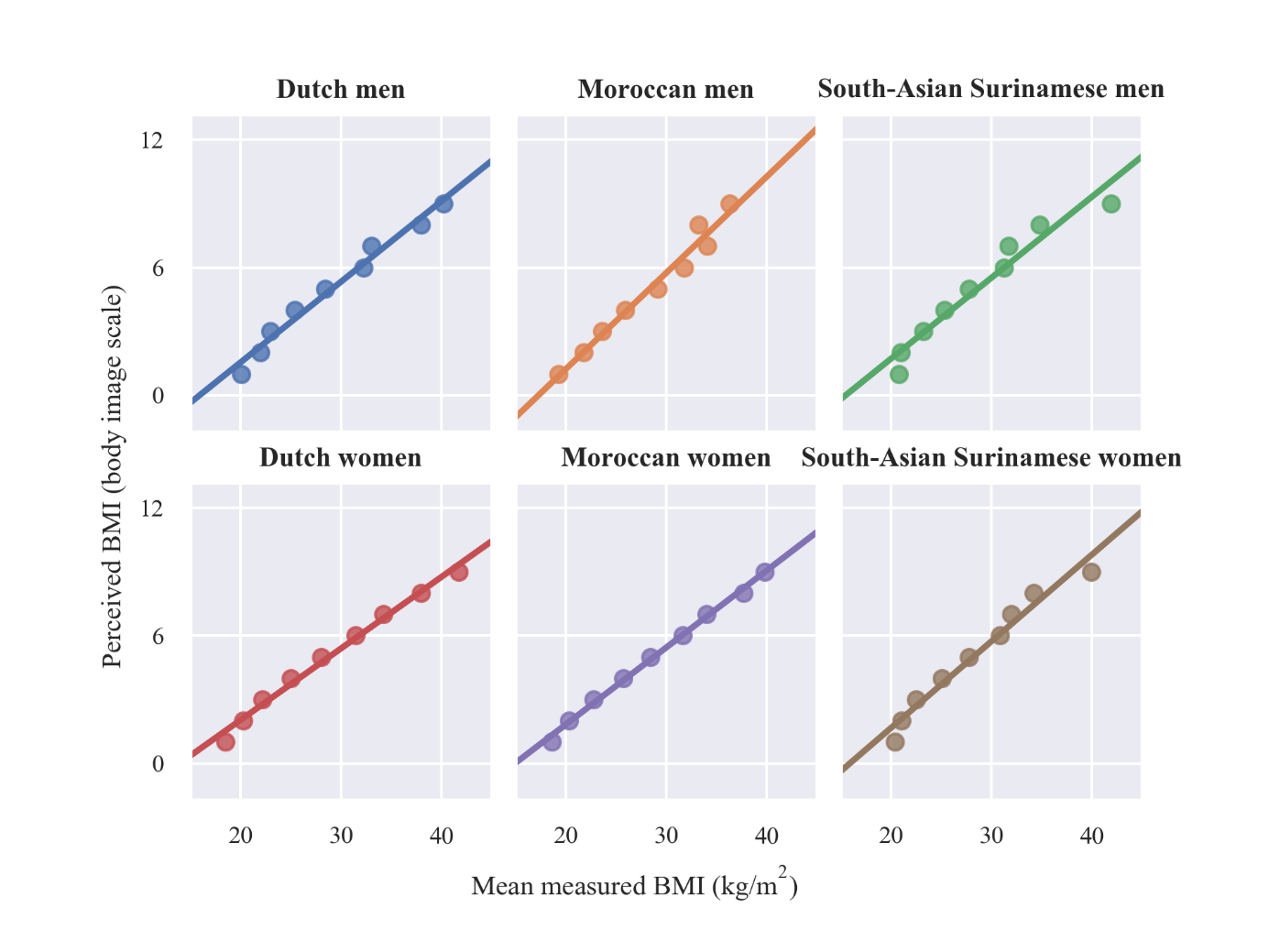


***Figure S1:* Visual representation of the calibration between perceived BMI as indicated by the body image scale and mean measured BMI for each group.** The solid line represents the linear regression line between the dependent variable *y* = perceived BMI as indicated by the body image scale, and the independent variable *x* = mean measured BMI. Accordingly, the dots represent the mean measured BMI corresponding to each of the nine images from the body image scale.

## Variables and equations

We give descriptions of the anthropometric and demographic variables, the energy balance variables, and their updating procedures below.

### Anthropometric and demographic variables

These variables are directly derived from the cohort data, where *H* (height) and *Age* are constants. *BMI* is calculated at each time step as

$${BMI}_{t+1}=\frac{W_{t+1}}{{H_{t=0}}^{2}},$$

(eq. (7) in the main manuscript),^3^ where *W* (weight) is updated according to eq. (6) in the main manuscript. *MedBMI* is the median *BMI* of the group (eq. (9)) and is also updated at every time step.

### Energy balance variables

We estimate *PAL* (physical activity level) at *t*=0 based on the SQUASH (Short QUestionnaire to ASsess Health-enhancing physical activity) validation study, where physical activity level (PAL) was determined for a sub-sample of the HELIUS study cohort (*n*=500) using a combined accelerometer and heart rate measurement device (Actiheart).^5^ This study shows that the mean PAL of individuals adhering to the Dutch physical activity norm is 1.65 (no units: expressed as *TDEE*/*BMR*, where *TDEE* and *BMR* refer to total daily energy intake and basal metabolic rate, respectively)^3^, whereas the mean PAL of individuals not adhering is 1.6.^5^ We extrapolate this cut-off point to all individuals in the study population, based on an available binary variable indicating whether an individual adheres to the norm. We round the PAL of adhering individuals up to 1.7 to correspond to accepted cut-off points^3^ of 1.40-1.69 for a sedentary/light activity lifestyle and 1.70-1.99 for an active/moderately active lifestyle. Accordingly, we assign a value of 1.7 to adhering individuals and 1.6 to those that do not. *PAL* is updated at each time step according to eq. (3) in the main manuscript. This equation also takes *PAL* at *t*=0 into account as a constant so that an individual’s value for *PAL* does not deviate significantly from initial their value to ensure that *PAL* evolves smoothly over time.

*BMR* is estimated at each time step based on the Schofield equation (eq. (8) in the main manuscript), which takes sex, *Age*, and *W* into account.^3^

For *TDEI* (total daily energy intake) and *TDEE* we assume that individuals are weight stable at *t*=0, thus we set the initial value of *TDEI* equal to the value of *TDEE*. After this, *TDEI* varies at each time step according to eq. (4) in the main manuscript, taking *TDEI* at *t*=0 into account as a constant to ensure that *TDEI* evolves smoothly over time. *TDEE* is calculated at each time step as

${TDEE}_{t+1}={BMR}_{t}\times{PAL}_{t+1}$,

(eq. (5)).^3^ We define *TDEE* based on *PAL* and *BMR*, so that it can be estimated independent of self-reported energy intake, which is often unreliable.^6^ *TMEI* (total monthly energy intake) and *TMEE* (total monthly energy expenditure) can be derived from their daily counterparts by multiplying them by 365/12 (average number of days/month) (eq. (6)).

*Rate_Weight gain_* (weight gain rate) and *Rate_Weight loss_* (weight loss rate) are calculated by converting *TMEI* and *TMEE* from kcal/month to kg/month (eq. (6)). Here we use the assumption that a surplus of 7,700 kilocalories accounts for a one kilogram increase in weight.^7,8^

### *Impact_HBonIIB_* as unique determinant of an individual’s behaviour with respect to *HB* and *Norm*

*Impact_HBonIIB_* (impact healthy BMI on individual ideal BMI) can be regarded as uniquely determining an individual’s behaviour with respect to *HB* (healthy BMI) and *Norm*, as we show using figure S2.

Here each blue dot represents 1-370 individuals that have that particular combination of distance *Norm* and *HB* and distance *Norm* and *IIB*. If *Impact_HBonIIB_* is close to 0, an individual does not let their *IIB* deviate from their perception of the norm, even if that perception of the norm is far from healthy. Conversely, if *Impact_HBonIIB_* is close to 1, the more equal *IIB* and *HB* are for an individual, i.e. the more that individual adapts what they strive for based on what is healthy, regardless of their perception of the norm. Accordingly, when we plot the regression through the origin line in eq. (1), the line for

${Impact}_{HBonIIB}=1$,

and the line for

${Impact}_{HBonIIB}=0$,

we can divide the separate regions of the plane defined by distance *Norm* and *HB* and distance *Norm* and *IIB* created by these lines according to different behaviours with respect to *HB* and *Norm*.


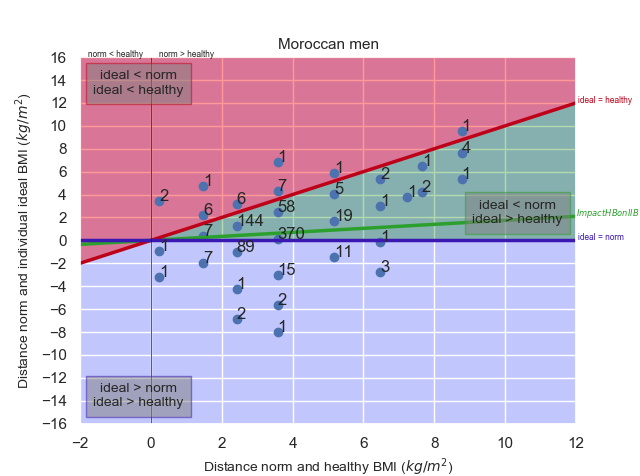

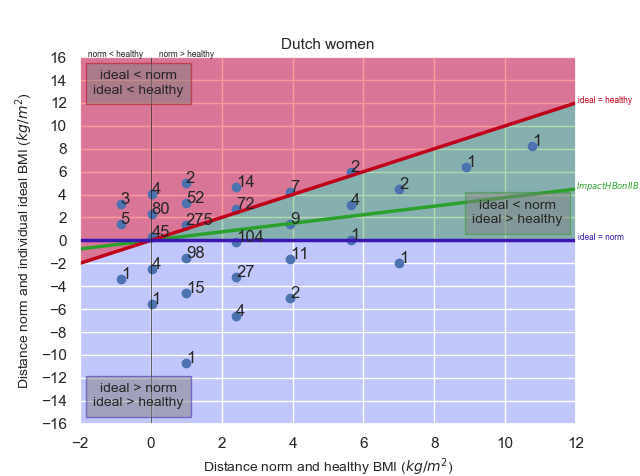


***Figure S2:* Regions corresponding to different behaviours with respect to *HB* and *Norm*.** Each blue dot represents 1-370 individuals that have that particular combination of distance *Norm* and *HB* and distance *Norm* and *IIB*. The distance between *Norm* and *HB* (x-axis) can only have a limited amount of values. This is because *Norm* depends on *MedBMI* and *SCIB*, where *SCIB* only has nine possible values (as it is estimated based on the body image scale), and because *HB* is the same for all individuals (22.5 kg/m^2^). The distance between *Norm* and *IIB* (y-axis) can only have a fairly limited amount of values because there are also only nine possible values for *IIB* (as it is estimated based on the body image scale).

As shown in figure S2, the values for *Impact_HBonIIB_* fall between 0 and 1 for each group. This implies that our assumptions are representative of the average behaviour of all individuals in a particular group, as depicted in the region shaded in green in figure S2. We show the behaviour for Moroccan men and Dutch women as they represent the groups that on an individual level behave the most and the least, respectively, according to our assumptions. For Moroccan men, we show that for most individuals their *IIB* is indeed lower than the norm, but higher than healthy. For Dutch women, even though the regression through the origin line falls in the region shaded in green, many individuals in fact strive to be thinner than both the norm and healthy, i.e. in the region shaded in red. Conversely, individuals that strive to be heavier than both the norm and healthy fall in the region shaded in green.

## From cross-sectional data to temporal dynamics

### Summary of previously proposed methods to generate pseudo-longitudinal data from cross-sectional data

Previous works have proposed methods to generate pseudo-longitudinal data from cross-sectional data by employing distance metrics, graph theoretical operations, and bootstrapping.^9–11^ The method of Peeling et al.^9^ is dependent on the ordering of the data points and an incorrect ordering can give spurious results. Tucker et al.^10^ proposed two similar methods for building pseudo time-series data from cross-sectional data based on constructing trajectories through the cross-sectional data space, starting at data points representing healthy cases and ending at data points representing diseased cases.^10^ Their first method is dependent on the size of the dataset and also a large difference between the number of diseased and healthy class labels and poor choices of the start-point and end-point will result in unrealistic trajectories and, therefore, poor temporal models.^10^ Their second method overcomes all of these issues except the reliance on the size of the dataset.^10^ Finally, the method proposed by Li et al.^11^ is also dependent on the ordering of the data points and the selection of the start-point and end-point.

This study highlights the value of integrating qualitative knowledge into the development of operational SDMs driven by cohort data. Our use of data-generating assumptions – as part of a newly derived, general methodology to design operational SDMs from cross-sectional data – makes our approach unaffected by the aforementioned methodological dependencies, though reliant on expert knowledge.

- **Basin-hopping algorithm**

We select the basin-hopping algorithm^12^ because this algorithm is generally efficient in finding the global optimum in the presence of large number of local optima. This algorithm is essentially a stochastic gradient-descent algorithm, where the height of the landscape represents the model fit error (cost). This strategy allows the algorithm to avoid getting stuck in a local optimum (minimum cost). If a randomly proposed move improves the solution (reduces cost) then it is always accepted. Otherwise, the algorithm accepts the move with some probability less than 1, which decreases exponentially with the increased cost of the move. Over time the probability of accepting such a ‘bad’ move decreases to zero to ensure convergence. We use the SciPy basin-hopping function (*scipy.optimize.basinhopping*)^13^ to perform the optimisation. We use the default values for all parameters in this function except the *take_step* and *niter_success* parameters. Since *Intent_EB_* (intent to change eating behaviour) and *Intent_PAB_* (intent to change physical activity behaviour) are of different scales, their step sizes should be different: *Intent_EB_* should have a larger step size compared to *Intent_PAB_*. To take this difference into account we define a custom step taking function and call it through the *take_step* parameter. We set the parameter *niter_success* to 50.

### Relation to energy landscape, approximation of local linearity, and data-generating assumptions

In the field of computational modelling, the underlying dynamics of certain complex systems can be operationalised in terms of an energy (or attractor) landscape, inspired by statistical mechanics and Hamiltonian dynamics.^14^ In short, an energy landscape assigns an energy value to a particular sequence of values for the variables (configuration). The energy value of a configuration is inversely related to its likelihood: a configuration with a lower energy value has a higher likelihood. The intuition is that the system configuration will explore slightly different configurations around its current state, and statistically is more likely to ‘choose’ a new configuration with lower energy (downhill as shown by the red arrows in figure S3). The stocks within an SDM represent the dimensions of the energy landscape, so in the present case the energy landscape has only one dimension (of the stock *W*). The configurations with the lowest energy value in the energy landscape are called the attractor points and they “correspond to stable equilibrium states” for the stocks.^15^


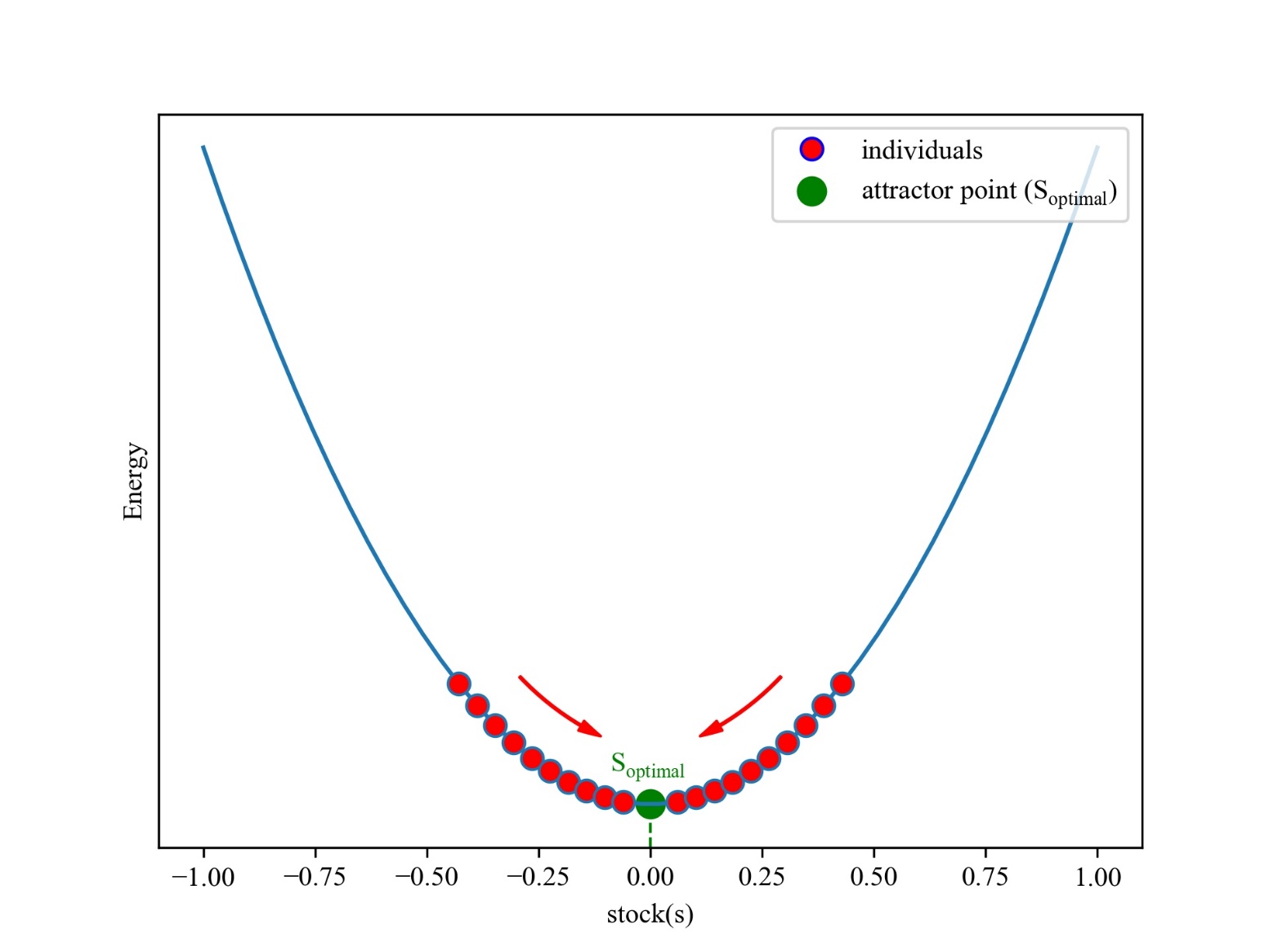


***Figure S3:* Energy landscape and behaviour of attractor for stock *s*.** The green dot represents the attractor point (*s_optimal_*) which is at stock value *s*=0 and the red dots represent the individuals, whose motion is towards the attractor point as indicated by the red arrows.

In the present study, we make the following assumptions about the energy landscape shown in figure S3 and the updating procedure of the stock value *s* for each individual:

**Assumption 1:** Over iterations *t* the stock value *s_t_ → s_optimal_* as *t → ∞*. In figure S3, this is represented by *s_optimal_*=0 (the green dot) as the attractor point. This means that we are modelling the expected value of individuals, i.e. no noise effects (random fluctuations) are modelled. As a consequence, the population variance should decrease monotonically over time.

**Assumption 2:** The energy landscape is convex and shallow. The latter is intended to make the instantaneous change per time unit small enough to warrant a linear approximation of causal effects (linear response theory), and to exclude pathological cases such as a delta function. Note that this linear approximation describes individual causal links; the response of the system over time may still be non-linear. This implies that each individual *i* is slowly moving towards the attractor point *s_optimal_*, and their displacement $s_{t+1}^{i}-s_{t}^{i}$ per time step is very small.

**Assumption 3:** All individuals (red dots in figure S3) are symmetrically distributed in the neighbourhood of *s_optimal_*. This allows identifying the position of *s_optimal_* as the center point of the point cloud of the individuals. Also, as a consequence, the displacements $s_{t+1}^{i}-s_{t}^{i}$ of all individuals are expected to add up to zero.

**Assumption 4:** Following from assumptions 1 and 2, for all individuals near *s_optimal_*,

$\lim_{t\to\infty} \frac{dE}{ds_{i}}\to0$,

which leads to our approximation of local linearity as explained in the following paragraph.

The updating procedure of each variable value for each individual at each time step is assumed to be a linear function of other variables in the system. The rationale is that non-linear behaviour cannot be inferred from data at a single time point. This approximation of local linearity is convenient for modelling short time spans and/or small changes of variables, as locally the slope of every smooth curve is approximately linear (figure S4). This implies a limited range of validity (variables may not vary strongly) and therefore we are only concerned with short-term dynamics in these proof-of-concept SDMs.


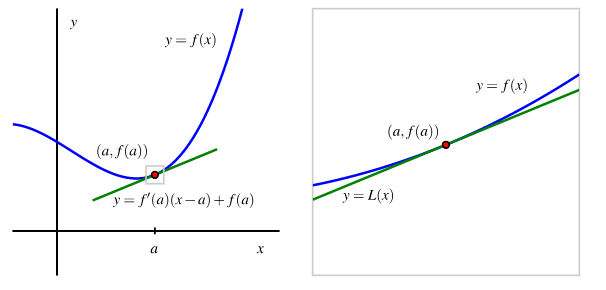


***Figure S4:* Illustration of local linearity.**^16^

In the present study, we model only the deterministic (conforming) tendency of individuals as driven by norms, i.e. always select the next downhill state in the energy landscape. Non-conforming events can be modelled as noise (occasional uphill movements) and can be used to explain the current variation around the attractor point (not everyone in the cohort is driven purely by norms) but here we focus only on the deterministic tendency to the attractor point. The consequence of this is that we underestimate the variation of values for *W* in our predictions (i.e. we assume that the weight of all individuals tends to the attractor point as driven by norms). This is acceptable in our case because the variation is not part of our research question, but it can easily be added in the future.

For each group, we are free to choose the ‘small’ average displacement in assumption 2 in the form of a parameter α. This parameter is defined as the average absolute displacement (for standardised data) expected in each time step.

We must first standardise the dataset to have zero mean and unit standard deviation (SD) because under the restriction of linear causal relations the only possible location for a single attractor is the zero vector $s=\vec{0}$. Standardisation occurs through applying the formula:

$$x_{standardised}=\frac{x_{original}-\mu}{\sigma}.$$

We standardise the state vector at *t*=0 as:

$$s_{t=0}^{standardised}=\frac{s_{t=0}-\mu_{s_{t=0}}}{\sigma_{s_{t=0}}}.$$

So, the state vector at *t*=0 has zero mean and unit SD. When we standardise the state vector at *t*=1, we want its SD to be less than 1 since the state vectors are moving towards the zero vector, thereby decreasing their variance. So, when we standardise the state vector at *t*=1, we divide by the SD of the state vector at *t*=0 and not by the SD of the state vector at *t*=1.

$$s_{t=1}^{standardised}=\frac{s_{t=1}-\mu_{s_{t=1}}}{\sigma_{s_{t=0}}}.$$

We can tune the value of α to match the amount of change that individuals on average experience in a certain time span, defining the time scale of the SDMs, which we choose to correspond to one month. The timescale of the model is determined by the choice of α but cannot be determined exactly without temporal data. Accordingly, α represents the average displacement, meaning that 1/α represents the number of time steps it takes for an individual to reach a close approximate of the attractor point. That is, if α=0.2, then an individual reaches a close approximate of the stable value for *W* as driven by norms in five time steps. As we cannot determine exactly what the attractor point *s*_∞_ of *W* is, for the purpose of approximating the value of α for each group, we assume the attractor point to be the average weight for that group. We have fitted α in such a way that an individual can on average lose 2 kg of weight per month (i.e. the amount of change that individuals on average experience in a certain time span), which is in line with literature addressing healthy weight loss.^17,18^ The role of α is to scale the curves along the x-axis, implying that the relative ordering of the curves remains the same for any value of α and accordingly making the exact value of α of secondary importance. The fitted α values are given in table S4.

***Table S4:* The fitted α value for each group.**

|  | Dutch men | Moroccan men | South-Asian Surinamese men | Dutch women | Moroccan women | South-Asian Surinamese women |
| --- | --- | --- | --- | --- | --- | --- |
| α | 0.159 | 0.169 | 0.172 | 0.150 | 0.157 | 0.162 |

We constrain the optimised parameters as *Intent_PAB_* > 0 and *Intent_EB_* < 0, which is based on eq. (3) and (4) for the SDMs.

$${PAL}_{t+1}={PAL}_{t=0}+{Intent}_{PAB}\times{{Discrepancy}_{BMIandIIB}}_{t}$$

$${TDEI}_{t+1}={TDEI}_{t=0}+{Intent}_{EB}\times{{Discrepancy}_{BMIandIIB}}_{t}$$

This constraint is a sanity check: *Intent_PAB_* and *Intent_EB_* represent the change that individuals make in physical activity behaviour (by changing their *PAL*) and in eating behaviour (by changing their *TDEI*) based on how much their *BMI* differs from their *IIB* (which is captured in *Discrepancy_BMIandIIB_* (discrepancy between BMI and individual ideal BMI). Accordingly:

- If *Discrepancy_BMIandIIB_* < 0, individuals think they should gain weight;
- if *Discrepancy_BMIandIIB_* = 0, individuals are satisfied with their weight;
- if *Discrepancy_BMIandIIB_* > 0, individuals think they should lose weight.

Based on *Discrepancy_BMIandIIB_*, we calculate the new values for *PAL* and *TDEI*, based on the hypothesis that individuals want to change their eating behaviour and their physical activity behaviour based on their (dis)satisfaction with their weight. We do this by multiplying *Discrepancy_BMIandIIB_* with *Intent_PAB_* to calculate the change in *PAL* to determine *PAL* at the next time step and by multiplying *Discrepancy_BMIandIIB_* with *Intent_EB_* to calculate the change in *TDEI* to determine *TDEI* at the next time step.

Therefore, *Intent_PAB_* should always be positive:

- If *Discrepancy_BMIandIIB_* < 0, this results in a negative change in *PAL* at the next time step (reduce physical activity and gain weight);
- if *Discrepancy_BMIandIIB_* = 0, the multiplication becomes zero (same physical activity and same weight);
- if *Discrepancy_BMIandIIB_* > 0, this results in a positive change in *PAL* at the next time step (increase physical activity and lose weight).

Similarly, we can say that *Intent_EB_* should always be negative:

- If *Discrepancy_BMIandIIB_* < 0, this results in a positive change in *TDEI* at the next time step (eat more and gain weight);
- if *Discrepancy_BMIandIIB_* = 0, the multiplication becomes zero (eat the same and keep the same weight);
- if *Discrepancy_BMIandIIB_* > 0, this results in a negative change in *TDEI* at the next time step (eat less and lose weight).

Using these assumptions we design a cost function that enables us to introduce a temporal aspect to the available cross-sectional data. That is, a cost function is used to constrain the parameter space to include only SDMs which satisfy these assumptions. The function implicitly places a next time point in the data for each individual (pseudo time-series) and evaluates how far off a given set of SDMs is from reproducing this second data point. Thus, even though we do not explicitly add a second data point to the cohort data – as our aim is to elucidate the *direction* an individual takes and not the exact value for each variable – we still evaluate whether a set of SDMs satisfies our assumptions. Namely, whether the dynamics that the SDMs exhibit are according to our assumptions. The definition of our cost function based on the aforementioned assumptions is described below.

### Cost function

Recall that the data are standardised to have zero mean and unit SD to conform with our assumption that the attractor point is the zero vector ($s_{\infty}=0$).

According to assumption 2, the displacement of each individual towards the attractor point $s_{\infty}=0$ is small. Accordingly, we assume that the short-term behaviour of each individual *i* can be approximated by a linear equation for displacement defined as:

$s_{t+1}^{i}=s_{t}^{i}+M\times s_{t}^{i}+c$. (1)

Here $s_{t+1}^{i}$ and $s_{t}^{i}$ are column vectors, *M* is a square matrix of dimension (size of vector *s*) and *c* is a constant column vector of dimension (size of vector *s*).

Now, we define a parameter α as the average absolute displacement expected in each time step. Since the data are standardised, it must be true that 0 < α < 1. So,

$\left\langle\left\| s_{t+1}^{i}-s_{t}^{i} \right\| \right\rangle_{i}=\left\langle\left\| M\times s_{t}^{i}+c \right\| \right\rangle_{i}=\alpha$. (2)

If we allow an SDM to run for unit time step, the displacement of each individual *i* can be defined as:

$\frac{\Delta s}{\Delta t}=\frac{s_{t+1}^{i}-s_{t}^{i}}{1}=s_{t+1}^{i}-s_{t}^{i}$. (3)

Now, according to assumption 2, the sum of the displacements of individuals at each time step equals zero since they are symmetrically distributed around the attractor point. So,

$\sum_{i} \left( s_{t+1}^{i}-s_{t}^{i} \right)=0$, (4)

$\sum_{i} M\times s_{t}^{i}+c=0$, (5)

$M\times\sum_{i} s_{t}^{i}+N\times c=0$. (6)

Also, the sum of the vectors representing the individuals, $\sum_{i} s_{t}^{i}$, equals the zero vector. Thus, we have,

$M\times0 + N\times c = 0,$ (7)

$c=0$. (8)

Therefore, having derived that $c=0$ as a consequence of our assumptions, equation (1) and equation (2) can be written as

$s_{t+1}^{i}=s_{t}^{i}+M\times s_{t}^{i},$ (9)

$\left\langle\left| \left| s_{t+1}^{i}-s_{t}^{i} \right| \right| \right\rangle_{i}=\left\langle\left| \left| M\times s_{t}^{i} \right| \right| \right\rangle_{i}=\alpha.$ (10)

The displacements of individuals should not only match an absolute value (α) but also ‘point’ towards the origin (zero vector). So, we assume that each individual *i* will simply reduce their norm, i.e. is multiplied by a constant $0<f_{i}<1$ for all *i*:

$\sqrt{\sum_{x} \left( s_{t}^{i}\left( x \right)\times f_{i} \right)^{2}}=\sqrt{\sum_{x} \left( s_{t}^{i}\left( x \right) \right)^{2}\times f_{i}^{2}}=f_{i}\times\sqrt{\sum_{x} \left( s_{t}^{i}\left( x \right) \right)^{2}}$. (11)

Each individual *i* will have their own constant $f_{i}$ which controls the individual’s speed with which he/she moves towards the zero vector and which is also the constant with which the individual’s norm is multiplied. The average over that should match with α. That is, in case of perfect movement to the origin, we have

$1-\left\langle f_{i} \right\rangle_{i}=\frac{\alpha}{\left\langle\left| \left| s_{t}^{i} \right| \right| \right\rangle_{i}}$ . (12)

To illustrate, if the model would rotate the individuals around the zero vector, i.e. along the circumference of a circle with the zero vector as the centre, then α may be matched but $\left\langle f_{i} \right\rangle_{i}=1$, since the norm of the individuals does not change. From now on we will denote $\left\langle f_{i} \right\rangle_{i}$ as *f*.

The first sub-cost function C_1_, controls the contraction of the individuals, i.e. the ratio of the observed norm at the time step *t+1* and the observed norm at the time step *t* should be close to *f*.

$C_{1}=\sqrt{\left\langle\left[ \frac{\left( \frac{\left\| s_{t+1}^{i} \right\|}{\left\| s_{t}^{i} \right\|} \right) - f}{\sigma\left( \frac{\left\| s_{t+1}^{i} \right\|}{\left\| s_{t}^{i} \right\|} \right)} \right]^{2} \right\rangle_{i}}$, (13)

where *σ(.)* denotes the standard deviation and is used to standardise C_1_.

The second sub-cost function C_2_ is based on the ratio of the contribution of *TDEI* and *PAL* on *W*, which is 75/25 according to expert knowledge (where 75% and 25% of the weight change are due to TDEI and PAL respectively). *TDEI* and *PAL* contribute to *W* through the weight gain rate (*wgr* in the equations below) and weight loss rate (*wlr* in the equations below), respectively. So, C_2_ ensures that the ratio of the change in *wgr* and *wlr* given in equation (14) is close to the ideal ratio of 75/25. Also, Δ*wgr* and Δ*wlr* should have opposite signs. If an individual loses weight then Δ*wgr* should be negative and Δ*wlr* should be positive; the opposite will happen if an individual gains weight. When calculating *wlr_t+1_*, we consider *BMR_t_* (i.e. we consider the same *BMR* for calculating *wlr_t_* and *wlr_t+1_*) and *PAL_t+1_* to take into account only the effect of *PAL* in *wlr*.

$ratio=\frac{\Delta wgr}{\Delta wlr}=\frac{\left| {wgr}_{t+1} \right|-\left| {wgr}_{t} \right|}{\left| {wlr}_{t+1} \right|-\left| {wlr}_{t} \right|}$, (14)

$C_{2}=\sqrt{\left\langle\left[ \frac{ratio-\left( -\frac{75}{25} \right)}{\sigma\left( ratio \right)} \right]^{2} \right\rangle_{i}}$, (15)

where *σ(.)* denotes the standard deviation and is used to standardise C_2_.

The final cost function, C, is a combination of the two sub-cost functions given in equations (13) and (15).

$$C=C_{1}+C_{2}$$

## Validation statements and weighting cohort data of the HELIUS study

### Validation statements

The validation statements (table S5) should be distinct from the expert knowledge used to construct the CLD and SDMs. In general this is difficult to ensure. Since the expert knowledge used for the CLD is of a ‘local’ nature (pertaining to a direct cause-and-effect) we therefore use ‘long-range’ statements which span multiple causal links in the SDMs, as well as statements which compare different ethnic and gender groups.

***Table S5:* Validation statements.**

|  | **Validation statement** | **Literature** | **Operationalisation** |
| --- | --- | --- | --- |
|  | There are no significant physiological differences between Dutch and South-Asian Surinamese men regarding the effect of physical activity on weight loss. | “Testing of sex and race/ethnicity interactions with adiposity and PA [physical activity] revealed no significant interactions.”^19^ | For the male groups, the effect of *PAL* on *BMI* should be of the same order of magnitude in the Dutch as the South-Asian Surinamese group. |
|  | There are no significant physiological differences between Dutch and Moroccan men regarding the effect of physical activity on weight loss. | Same as above.^19,20^ | For the male groups, the effect of *PAL* on *BMI* should be of the same order of magnitude in the Dutch as the Moroccan group. |
|  | There are no significant physiological differences between South-Asian Surinamese and Moroccan men regarding the effect of physical activity on weight loss. | Same as above.^19,20^ | For the male groups, the effect of *PAL* on *BMI* should be of the same order of magnitude in the South-Asian Surinamese as the Moroccan group. |
|  | There are no significant physiological differences between Dutch and South-Asian Surinamese women regarding the effect of physical activity on weight loss. | Same as above.^19,20^ | For the female groups, the effect of *PAL* on *BMI* should be of the same order of magnitude in the Dutch as the South-Asian Surinamese group. |
|  | There are no significant physiological differences between Dutch and Moroccan women regarding the effect of physical activity on weight loss. | Same as above.^19,20^ | For the female groups, the effect of *PAL* on *BMI* should be of the same order of magnitude in the Dutch as the Moroccan group. |
|  | There are no significant physiological differences between South-Asian Surinamese and Moroccan women regarding the effect of physical activity on weight loss. | Same as above.^19,20^ | For the female groups, the effect of *PAL* on *BMI* should be of the same order of magnitude in the South-Asian Surinamese as the Moroccan group. |
|  | There are no significant physiological differences between Dutch men and women regarding the effect of physical activity on weight loss. | Same as above.^19,20^ | For the Dutch group, the effect of *PAL* on *BMI* should be of the same order of magnitude in the male as in the female group. |
|  | There are no significant physiological differences between South-Asian Surinamese men and women regarding the effect of physical activity on weight loss. | Same as above.^19,20^ | For the South-Asian Surinamese group, the effect of *PAL* on *BMI* should be of the same order of magnitude in the male as in the female group. |
|  | There are no significant physiological differences between Moroccan men and women regarding the effect of physical activity on weight loss. | Same as above.^19,20^ | For the Moroccan group, the effect of *PAL* on *BMI* should be of the same order of magnitude in the male as in the female group. |
|  | Norms tend to have a larger effect on Moroccan than on Dutch men, as Moroccan culture is regarded as being more collectivistic than Dutch culture. | “All three minority cultural groups [Moroccan, South-Asian Surinamese and one other] are characterized as collectivistic.^21^ For example, studies of child-rearing practices indicated that conformity is a very important goal of parenting among […] Moroccan, and South Asian-Surinamese parents, whereas Dutch parents focus much more on autonomy skills.^22,23^”^24^  “Conformity to social norms is more likely in Eastern, collectivistic cultures than in Western, independent cultures.”^25^ | For the male groups, (1 – *Impact_HBonIIB_*), representing the impact of *Norm* on *IIB*, should be higher in the Moroccan than in the Dutch group. |
|  | Norms tend to have a larger effect on South-Asian Surinamese than on Dutch men, as South-Asian Surinamese culture is regarded as being more collectivistic than Dutch culture. | Same as above.^22–25^ | For the male groups, (1 – *Impact_HBonIIB_*), representing the impact of *Norm* on *IIB*, should be higher in the South-Asian Surinamese than in the Dutch group. |
|  | Norms tend to have a larger effect on Moroccan than on South-Asian Surinamese men, as Moroccan culture is regarded as being more collectivistic than South-Asian Surinamese culture. | “All three minority cultural groups [Moroccan, South-Asian Surinamese and one other] are characterized as collectivistic.^21^ For example, studies of child-rearing practices indicated that conformity is a very important goal of parenting among […] Moroccan, and South Asian-Surinamese parents, whereas Dutch parents focus much more on autonomy skills.^22,23^”^24^  “Conformity to social norms is more likely in Eastern, collectivistic cultures than in Western, independent cultures.”^25^  “The assumption […] [is] that the longer the duration of residence, the more assimilated a person is to the Dutch culture […].”^26^  Characteristics of the HELIUS study participants by ethnicity showed that on average South-Asian Surinamese have a longer residence duration in the Netherlands (33.1 years) than Moroccans (29.0 years).^26^  In addition, another study using the HELIUS cohort data showed that 65.5% of the Moroccans had a “strong ethnic identity” score, as compared to 51.8% of the South-Asian Surinamese.^27^ Similarly, this study showed that 39.7% of the Moroccans had more than four “same-ethnic friends”, as compared to 29.3% of the South-Asian Surinamese.^27^ | For the male groups, (1 – *Impact_HBonIIB_*), representing the impact of *Norm* on *IIB*, should be higher in the Moroccan than in the South-Asian Surinamese group. |
|  | Norms tend to have a larger effect on Moroccan than on Dutch women, as Moroccan culture is regarded as being more collectivistic than Dutch culture. | “All three minority cultural groups [Moroccan, South-Asian Surinamese and one other] are characterized as collectivistic.^21^ For example, studies of child-rearing practices indicated that conformity is a very important goal of parenting among […] Moroccan, and South Asian-Surinamese parents, whereas Dutch parents focus much more on autonomy skills.^22,23^”^24^  “Conformity to social norms is more likely in Eastern, collectivistic cultures than in Western, independent cultures.”^25^ | For the female groups, (1 – *Impact_HBonIIB_*), representing the impact of *Norm* on *IIB*, should be higher in the Moroccan than in the Dutch group. |
|  | Norms tend to have a larger effect on South-Asian Surinamese than on Dutch women, as South-Asian Surinamese culture is regarded as being more collectivistic than Dutch culture. | Same as above.^22–25^ | For the female groups, (1 – *Impact_HBonIIB_*), representing the impact of *Norm* on *IIB*, should be higher in the South-Asian Surinamese than in the Dutch group. |
|  | Norms tend to have a larger effect on Moroccan than on South-Asian Surinamese women, as Moroccan culture is regarded as being more collectivistic than South-Asian Surinamese culture. | “All three minority cultural groups [Moroccan, South-Asian Surinamese and one other] are characterized as collectivistic.^21^ For example, studies of child-rearing practices indicated that conformity is a very important goal of parenting among […] Moroccan, and South Asian-Surinamese parents, whereas Dutch parents focus much more on autonomy skills.^22,23^”^24^  “Conformity to social norms is more likely in Eastern, collectivistic cultures than in Western, independent cultures.”^25^  “The assumption […] [is] that the longer the duration of residence, the more assimilated a person is to the Dutch culture […].”^26^  Characteristics of the HELIUS study participants by ethnicity showed that on average South-Asian Surinamese have a longer residence duration in the Netherlands (33.1 years) than Moroccans (29.0 years).^26^  In addition, another study using the HELIUS cohort data showed that 65.5% of the Moroccans had a “strong ethnic identity” score, as compared to 51.8% of the South-Asian Surinamese.^27^ Similarly, this study showed that 39.7% of the Moroccans had more than four “same-ethnic friends”, as compared to 29.3% of the South-Asian Surinamese.^27^ | For the female groups, (1 – *Impact_HBonIIB_*), representing the impact of *Norm* on *IIB*, should be higher in the Moroccan than in the South-Asian Surinamese group. |
|  | Dutch women tend to be more prone to be influenced by norms than Dutch men. | “Taken together, this means that, at least when they are being observed by others, men are likely to hold their ground, act independently, and refuse to conform, whereas women are more likely to conform to the opinions of others in order to prevent social disagreement.”^25^ | For the Dutch group, (1 – *Impact_HBonIIB_*), representing the impact of *Norm* on *IIB*, should be higher in the female than in the male group. |
|  | South-Asian Surinamese women tend to be more prone to be influenced by norms than South-Asian Surinamese men. | Same as above.^25^ | For the South-Asian Surinamese group, (1 – *Impact_HBonIIB_*), representing the impact of *Norm* on *IIB*, should be higher in the female than in male group. |
|  | Moroccan women tend to be more prone to be influenced by norms than Moroccan men. | Same as above.^25^ | For the Moroccan group, (1 – *Impact_HBonIIB_*), representing the impact of *Norm* on *IIB*, should be higher in the female than in the male group. |
|  | The intent to change physical activity behaviour tends to be greater in Dutch than in Moroccan men, as physical activity is more embedded in Dutch than in Moroccan culture. | “Studies indicate that ethnic minority groups [in Europe] are both less active and more sedentary than the majority population […].”^28^  “In the Netherlands, ethnic minority groups are generally less physically active and rate their own health poorer compared to ethnic Dutch.”^29^  “Some comparative analyses across countries in the Arab region and outside it have reported that Muslim countries were more likely to be physically inactive, and seemed to suggest that religion constitutes an obstacle to physical activity^30^.”^31^ | For the male groups, *Intent_PAB_* should be higher in the Dutch than in the Moroccan group. |
|  | The intent to change physical activity behaviour tends to be greater in Dutch than in South-Asian Surinamese men, as physical activity is more embedded in Dutch than in South-Asian Surinamese culture. | “Studies indicate that ethnic minority groups [in Europe] are both less active and more sedentary than the majority population […].”^28^  “In the Netherlands, ethnic minority groups are generally less physically active and rate their own health poorer compared to ethnic Dutch.”^29^  “[…] the effect of ethnicity on physical activity was mediated through perceived behaviour control (Asians less active than Caucasians).”^32^  “Results [of a focus group with members from a Surinamese Hindustani community in the Netherlands] showed cultural implications that might affect the effectiveness of health education messages: karma has a role in explaining the onset of illness, traditional eating habits are perceived as difficult to change, and PA [physical activity] was generally disliked.”^33^  “Participants [of South-Asian Surinamese ethnicity] reported that sports and other forms of PA [physical activity] are not encouraged within their communities. Children are not encouraged to spend time being physically active as it is seen as interfering with time that could be invested in studying or working. Among adults, taking time to exercise was perceived as interfering with other, more important, obligations such as spending time with family or fulfilling social obligations.”^34^  “Socio-cultural beliefs appeared to influence participants’ [of South-Asian Surinamese ethnicity] perceptions regarding PA. In Suriname people are physically active as part of their daily routines: working on the land, household tasks and transportation. Hence participants explained that activity during leisure time or to achieve physical fitness was an unfamiliar concept.”^34^  “The climate in the Netherlands was mentioned as being an important barrier for engaging in PA [physical activity] [for the South-Asian Surinamese]. Cold and rain were stated as reasons to refrain from outdoor activities and many participants mentioned only being physically active in the summer. Ready availability of public transport or car ownership was often stated as being deterrents to active forms of transport, particularly given climatic conditions.”^34^ | For the male groups, *Intent_PAB_* should be higher in the Dutch than in the South-Asian Surinamese group. |
|  | The intent to change physical activity behaviour tends to be greater in Dutch than in Moroccan women, as physical activity is more embedded in Dutch than in Moroccan culture. | “Studies indicate that ethnic minority groups [in Europe] are both less active and more sedentary than the majority population […].”^28^  “In the Netherlands, ethnic minority groups are generally less physically active and rate their own health poorer compared to ethnic Dutch.”^29^  “Some comparative analyses across countries in the Arab region and outside it have reported that Muslim countries were more likely to be physically inactive, and seemed to suggest that religion constitutes an obstacle to physical activity^30^.”^31^  “Results from women in both locations [Moroccan migrant women and their non-migrant compatriots that live in Morocco] indicate a general lack of knowledge regarding appropriate physical activity.”^35^ | For the female groups, *Intent_PAB_* should be higher in the Dutch than in the Moroccan group. |
|  | The intent to change physical activity behaviour tends to be greater in Dutch than in South-Asian Surinamese women, as physical activity is more embedded in Dutch than in South-Asian Surinamese culture. | “Studies indicate that ethnic minority groups [in Europe] are both less active and more sedentary than the majority population […].”^28^  “In the Netherlands, ethnic minority groups are generally less physically active and rate their own health poorer compared to ethnic Dutch.”^29^  “[…] the effect of ethnicity on physical activity was mediated through perceived behaviour control (Asians less active than Caucasians).”^32^  “Results [of a focus group with members from a Surinamese Hindustani community in the Netherlands] showed cultural implications that might affect the effectiveness of health education messages: karma has a role in explaining the onset of illness, traditional eating habits are perceived as difficult to change, and PA [physical activity] was generally disliked.”^33^  “Participants [of South-Asian Surinamese ethnicity] reported that sports and other forms of PA [physical activity] are not encouraged within their communities. Children are not encouraged to spend time being physically active as it is seen as interfering with time that could be invested in studying or working. Among adults, taking time to exercise was perceived as interfering with other, more important, obligations such as spending time with family or fulfilling social obligations.”^34^  “Socio-cultural beliefs appeared to influence participants' [of South-Asian Surinamese ethnicity] perceptions regarding PA. In Suriname people are physically active as part of their daily routines: working on the land, household tasks and transportation. Hence participants explained that activity during leisure time or to achieve physical fitness was an unfamiliar concept.”^34^  “The climate in the Netherlands was mentioned as being an important barrier for engaging in PA [physical activity] [for the South-Asian Surinamese]. Cold and rain were stated as reasons to refrain from outdoor activities and many participants mentioned only being physically active in the summer. Ready availability of public transport or car ownership was often stated as being deterrents to active forms of transport, particularly given climatic conditions.”^34^ | For the female groups, *Intent_PAB_* should be higher in the Dutch than in the South-Asian Surinamese group. |
|  | The intent to change physical activity behaviour tends to be greater in Dutch women than in Dutch men. | “In the Netherlands, 20.0% of the population aged 15 years and over were insufficiently active (men 23.7% and women 16.4%), according to estimates generated for 2008 by WHO.^36^”^37^ | For the Dutch group, *Intent_PAB_* should be higher in the female than in the male group. |
|  | The intent to change physical activity behaviour tends to be greater in South-Asian Surinamese men than in South-Asian Surinamese women. | “Opinions on sex roles among these three minority groups [Moroccan, South-Asian Surinamese and one other] are more traditional when compared to the majority Dutch^38^.”^24^  “[…] for many women in South Asian families, any spare time is expected to be utilized to help benefit the family […].^39^ In certain households, male members do not want their wives to go outside and exercise.^39–42^ For instance, the female figure in the home is expected to be available in order to provide food for the male when he wants it, making it hard or even unacceptable for women to leave the home.^39,43^ These views are often upheld by others in the community. As a result, a woman who is seen exercising or even going for a walk outside may be looked down upon, as is her family.^44^”^45^  “Although it is changing, traditionally, South Asian culture has emphasized physical separation between men and women. Hence, in regards to physical activity, many women only feel comfortable in female specific facilities and feel that it is culturally inappropriate to exercise around men. The absence of separation in most exercise facilities and gyms often dissuades South Asian females from taking advantage of exercise programs or joining a health club.^41,44^”^45^  “They explained that PA [physical activity] is more likely to be accepted in young [South-Asian Surinamese] men, whereas [South-Asian Surinamese] women are traditionally not encouraged to engage in activities outside the home.”^34^ | For the South-Asian Surinamese group, *Intent_PAB_* should be higher in the male than in the female group. |
|  | The intent to change physical activity behaviour tends to be greater in Moroccan men than in Moroccan women. | “Opinions on sex roles among these three minority groups [Moroccan, South-Asian Surinamese and one other] are more traditional when compared to the majority Dutch^38^.”^24^  “Boys [of Moroccan nationality] were eight times more likely to meet the recommendation for at least 60 min of moderate to vigorous physical activity per day than girls […].”^46^  “The accumulation of traditional roles [in Moroccan culture], the most significant being the role of wife, contributes to a woman's obesity risk for several reasons. First, the expected activities associated with the role of a wife are located exclusively in the household and are often centred on food preparation. Secondly, prior to marriage, a woman may have more leisure time to participate in physical activity or other beneficial health activities.”^47^ | For the Moroccan group, *Intent_PAB_* should be higher in the male than in the female group. |
|  | The intent to change eating behaviour tends to be greater in Dutch than in Moroccan men, as eating behaviour is more important in Moroccan than in Dutch culture. | “Apart from social events, (social) habits within the family were perceived as constraints to healthy eating. Various […] Moroccan women reported cooking several dishes per day to satisfy each family member’s food preferences. […] These women want to please all family members to cherish the family relationship, and individual goals are subordinate to the collective well-being. Eating is not an individual act but often performed with others; and competing values, like, in this case, caring for the family, can limit a person’s willingness to comply with health recommendations.^48^ When individuals change their eating habits, this can also affect others who take part in the act of eating […] Dutch families, however, seemed to be more likely to support the person’s change in lifestyle, whereas […] Moroccan participants reported being less supported or to give less support to change eating habits. Eating is often not an individual decision but an activity that is deeply embedded in social life and also depends on the social practices of others. Social practices are shaped by social norms and values and by opportunities emerging in specific social situations. In daily life, individuals interact in specific social situations that produce health-related behaviours.^49^”^50^  “Among Dutch residents of […] Moroccan migrant origin, the central role of food in culture coupled with the changes that come about as a result of migration create an environment of abundance that can lead to overeating, which may impact energy balance and overweight development.”^51^ | For the male groups, *Intent_EB_* should be higher in the Dutch than in the Moroccan group. |
|  | The intent to change eating behaviour tends to be greater in Dutch than in South-Asian Surinamese men, as eating behaviour is more important in South-Asian Surinamese than in Dutch culture. | “Participants offered the opinion that their traditional foods are unhealthy and expressed the idea that ‘tradition’ and ‘health’ are not compatible with each other. However, the more unhealthy characteristics of traditional foods were also described as contributing to their flavour. Using alternative ingredients (such as brown rice) or alternative preparation methods were perceived as negatively influencing the taste of foods. […] Food is an important part of social interactions within the South-Asian Surinamese culture.”^34^ | For the male groups, *Intent_EB_* should be higher in the Dutch than in the South-Asian Surinamese group. |
|  | The intent to change eating behaviour tends to be greater in Dutch than in Moroccan women, as eating behaviour is more important in Moroccan than in Dutch culture. | “Apart from social events, (social) habits within the family were perceived as constraints to healthy eating. Various […] Moroccan women reported cooking several dishes per day to satisfy each family member’s food preferences. […] These women want to please all family members to cherish the family relationship, and individual goals are subordinate to the collective well-being. Eating is not an individual act but often performed with others; and competing values, like, in this case, caring for the family, can limit a person’s willingness to comply with health recommendations.^48^ When individuals change their eating habits, this can also affect others who take part in the act of eating […] Dutch families, however, seemed to be more likely to support the person’s change in lifestyle, whereas […] Moroccan participants reported being less supported or to give less support to change eating habits. Eating is often not an individual decision but an activity that is deeply embedded in social life and also depends on the social practices of others. Social practices are shaped by social norms and values and by opportunities emerging in specific social situations. In daily life, individuals interact in specific social situations that produce health-related behaviours.^49^”^50^  “In focus groups with migrant women in Amsterdam, participants attributed overweight to traditional Moroccan foods and food culture.”^35^  “Among Dutch residents of […] Moroccan migrant origin, the central role of food in culture coupled with the changes that come about as a result of migration create an environment of abundance that can lead to overeating, which may impact energy balance and overweight development.”^51^ | For the female groups, *Intent_EB_* should be higher in the Dutch than in the Moroccan group. |
|  | The intent to change eating behaviour tends to be greater in Dutch than in South-Asian Surinamese women, as eating behaviour is more important in South-Asian Surinamese than in Dutch culture. | “Participants offered the opinion that their traditional foods are unhealthy and expressed the idea that ‘tradition’ and ‘health’ are not compatible with each other. However, the more unhealthy characteristics of traditional foods were also described as contributing to their flavor. Using alternative ingredients (such as brown rice) or alternative preparation methods were perceived as negatively influencing the taste of foods. […] Food is an important part of social interactions within the Surinamese SA [South-Asian] culture.”^34^ | For the female groups, *Intent_EB_* should be higher in the Dutch than in the South-Asian Surinamese group. |
|  | The intent to change eating behaviour tends to be greater in Dutch women than in Dutch men. | “Dieting was more common among [Dutch] women than [Dutch] men […].”^52^ | For the Dutch group, *Intent_EB_* should be higher in the female than in the male group. |
|  | The intent to change eating behaviour tends to be greater in Moroccan women than in Moroccan men. | In the project “Dietary habits among young Dutch people of Turkish and Moroccan origin: a problem analysis and needs assessment for the development of appropriate nutrition interventions”, the results of around 10 focus group discussions with 100 Moroccan men and women showed that mostly women are interested in food and health.^53^ | For the Moroccan group, *Intent_EB_* should be higher in the female than in the male group. |
|  | The intent to change eating behaviour tends to be greater in South-Asian Surinamese women than in South-Asian Surinamese men. | “As might be expected, [South-Asian Surinamese] women seemed to pay more attention to their diet than [South-Asian Surinamese] men; many women stated that they were very conscious about health by, for example, paying attention to the amount of oil used during food preparation and making sure to include vegetables in meals.”^34^ | For the South-Asian Surinamese group, *Intent_EB_* should be higher in the female than in the male group. |

For **validation statements 1-9**, we test whether the effect of *PAL* on *BMI* is of the same order of magnitude for different pairs of SDMs. To calculate the effect of *PAL* on *BMI*, we increase *PAL* by 10% and determine the stable *MedBMI* *(*denoted as ${MedBMI}_{10\% increased PAL}\left( stable \right)$ in the following equation). Then we determine the effect of *PAL* on *BMI* as the percentage difference between ${MedBMI}_{10\% increased PAL}\left( stable \right)$ and the stable *MedBMI* corresponding to the original *PAL* (${MedBMI}_{original}\left( stable \right)$) divided by ${MedBMI}_{original}\left( stable \right)$. This is represented by the following equation:

$${Effect}_{PALonBMI}=\frac{{MedBMI}_{10\% increased PAL}\left( stable \right)-{MedBMI}_{original}\left( stable \right)}{{MedBMI}_{original}\left( stable \right)}\times100.$$

Then, to determine the order of magnitude of the *Effect_PALonBMI_*, we take the logarithm (base 10) of this value for each group. Validation statements 1-9 state that the *Effect_PALonBMI_* is of the same order of magnitude in the respective groups mentioned in each of the statements. To verify this, we take the rounded ratio of the logarithmic values of the *Effect_PAL on BMI_* for the respective groups and if this rounded ratio equals 1 we determine that the values of *Effect_PALonBMI_* for the respective groups are of the same order.

For **validation statements 10-18**, we compare the effect of *Norm* on *IIB* among different pairs of SDMs. *IIB* is a weighted average of *HB* and *Norm*, where the parameter *Impact_HBonIIB_* determines the relative weight of each. A larger *Impact_HBonIIB_* implies a larger effect of *HB* on *IIB* and as a consequence a lower effect of *Norm* on *IIB*. Hence, to estimate the effect of *Norm* on *IIB*, we directly compare the impact of *Norm* on *IIB*, as represented by (1 – *Impact_HBonIIB_*), between the respective pair of SDMs.

For **validation statements 19-25**, we compare the absolute values of *Intent_PAB_* for the different pairs of SDMs specified in the statements.

Similarly, for **validation statements 26-29**, we compare the absolute values of *Intent_EB_* for the different pairs of SDMs specified in the statements. For **validation statements 30-32**, before comparing their absolute values, we scale the *Intent_EB_* values for the male and female groups based on the given that when adhering to a healthy, balanced diet, a man needs around 2,500 kcal per day whereas a woman needs around 2,000 kcal per day to maintain his/her weight.^54–56^ To account for this discrepancy in required calorie intake to maintain weight, we make the *Intent_EB_* values for the male and female groups comparable, as we seek to determine the *relative* change in eating behaviour in a particular group. We accordingly multiply the *Intent_EB_* values of the male groups with the factor 2,000/2,500, to obtain an estimate of the *Intent_EB_* values of the male groups if they were to need 2,000 kcal per day to maintain weight, to make them comparable with the values of the female groups.

### Weighting cohort data of the HELIUS study

To verify that the study population is representative of the population of the Netherlands (with its different ethnic groups), as most of our supporting literature refers to the population of the Netherlands, we also see how many validation statements are correct after we weight the cohort data according to the age distribution of the population of the Netherlands corresponding to each group.

Hence, to make the comparisons between groups as described in the statements more reliable, we adjust the age distribution of the cohort data to reflect the age distribution of the population of the Netherlands, based on a dataset that reflects the population of the Netherlands as stratified by age, migration background, sex, and region.^57^

For this purpose, we divide the cohort data into five age groups: 18-29, 30-39, 40-49, 50-59, and 60+. In the weight adjustment technique, if a particular age group is under-represented then we add individuals to that age group in the cohort data based on the fraction of individuals in that age group in the population of the Netherlands. Similarly, if a particular age group is over-represented then we remove individuals from that age group in the cohort data based on the fraction of individuals in that age group in the population of the Netherlands.

To gain more insight into how weighting might adjust the validation score, we also perform this weight adjustment technique to make the age distribution of the cohort data reflect the age distribution of the population of Amsterdam, based on a dataset that reflects the population of Amsterdam as stratified by age and migration background.^58^ The age distributions of the cohort data, the population of the Netherlands, and the population of Amsterdam are given in figure S5.

We give the results of the number of validation statements satisfied by the SDMs (out of 32 validation statements in total) according to the different age distributions in table S6. The number of validation statements satisfied by the SDMs with the weight adjusted cohort data are given in the form of mean ± SD because the weight adjustment technique involves the addition or removal of random individuals in a particular age group based on the fraction of individuals in that age group in the population (of the Netherlands and Amsterdam). To take into account the variance in the sampling distribution, we repeat the weight adjustment process 1,000 times for each population (of the Netherlands and Amsterdam), from which we get a distribution of the number of validation statements satisfied.

Secondly, we show the results of which particular validation statements are satisfied by the SDMs under which age weighting adjustments in table S7. Here, we provide the majority vote (in terms of *Yes/No*) of the 1,000 times we repeat the adjustment process for each population (of the Netherlands and Amsterdam) for each statement.

For the age distribution reflecting the population of the Netherlands, the same set of validation statements is correct as for the original age distribution of the cohort data. For the age distribution reflecting the population of Amsterdam, validation statement 13 is correct (shown in **bold** in table S7) whereas it is not for the original age distribution of the cohort data. Taking these results together, we conclude that weighting does not considerably alter the behaviour of the SDMs, implying that the cohort data are representative for the functioning of this system.


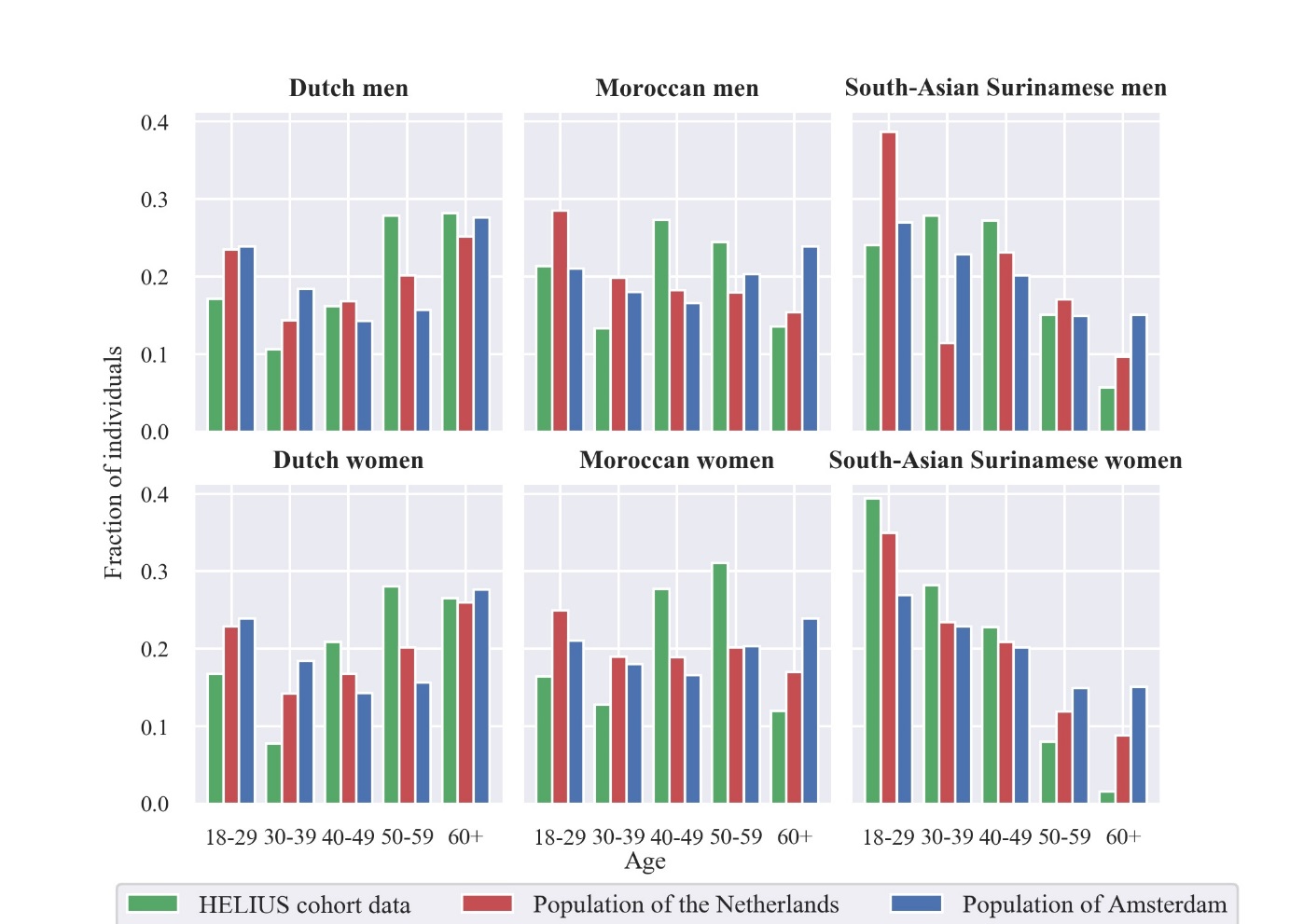


***Figure S5:* The age distributions of the cohort data of the HELIUS study, the population of the Netherlands, and the population of Amsterdam.**

***Table S6:* Number of validation statements (out of 32) satisfied by the SDMs according to the original age distribution of the cohort data of the HELIUS study, reflecting the age distribution of the population of the Netherlands, and reflecting the age distribution of the population of Amsterdam.**

| Number of validation statements (out of 32) satisfied by the SDMs | The original age distribution of the cohort data of the HELIUS study | 20 |
| --- | --- | --- |
|  | Reflecting the age distribution of the population of the Netherlands, mean ± SD | 20 ± 0.65 |
|  | Reflecting the age distribution of the population of Amsterdam, mean ± SD | 21 ± 0.35 |

***Table S7:* Behaviours exhibited by the SDMs according to the actual cohort data of the HELIUS study, reflecting the age distribution of the population of the Netherlands, and reflecting the age distribution of the population of Amsterdam.**

|  | **Operationalisation** | **Behaviour exhibited by the SDMs (*Yes/No)*** | | |
| --- | --- | --- | --- | --- |
|  |  | The original age distribution of the cohort data of the HELIUS study | Reflecting the age distribution of the population of the Netherlands, majority vote out of 1,000 | Reflecting the age distribution in the population of Amsterdam, majority vote out of 1,000 |
|  | For the male groups, the effect of *PAL* on *BMI* should be of the same order of magnitude in the Dutch as the South-Asian Surinamese group. | Yes | Yes | Yes |
|  | For the male groups, the effect of *PAL* on *BMI* should be of the same order of magnitude in the Dutch as the Moroccan group. | Yes | Yes | Yes |
|  | For the male groups, the effect of *PAL* on *BMI* should be of the same order of magnitude in the South-Asian Surinamese as the Moroccan group. | Yes | Yes | Yes |
|  | For the female groups, the effect of *PAL* on *BMI* should be of the same order of magnitude in the Dutch as the South-Asian Surinamese group. | Yes | Yes | Yes |
|  | For the female groups, the effect of *PAL* on *BMI* should be of the same order of magnitude in the Dutch as the Moroccan group. | Yes | Yes | Yes |
|  | For the female groups, the effect of *PAL* on *BMI* should be of the same order of magnitude in the South-Asian Surinamese as the Moroccan group. | Yes | Yes | Yes |
|  | For the Dutch group, the effect of *PAL* on *BMI* should be of the same order of magnitude in the male as in the female group. | Yes | Yes | Yes |
|  | For the South-Asian Surinamese group, the effect of *PAL* on *BMI* should be of the same order of magnitude in the male as in the female group. | Yes | Yes | Yes |
|  | For the Moroccan group, the effect of *PAL* on *BMI* should be of the same order of magnitude in the male as in the female group. | Yes | Yes | Yes |
|  | For the male groups, (1 – *Impact_HBonIIB_*), representing the impact of *Norm* on *IIB*, should be higher in the Moroccan than in the Dutch group. | Yes | Yes | Yes |
|  | For the male groups, (1 – *Impact_HBonIIB_*), representing the impact of *Norm* on *IIB*, should be higher in the South-Asian Surinamese than in the Dutch group. | No | No | No |
|  | For the male groups, (1 – *Impact_HBonIIB_*), representing the impact of *Norm* on *IIB*, should be higher in the Moroccan than in the South-Asian Surinamese group. | Yes | Yes | Yes |
|  | For the female groups, (1 – *Impact_HBonIIB_*), representing the impact of *Norm* on *IIB*, should be higher in the Moroccan than in the Dutch group. | No | No | **Yes** |
|  | For the female groups, (1 – *Impact_HBonIIB_*), representing the impact of *Norm* on *IIB*, should be higher in the South-Asian Surinamese than in the Dutch group. | No | No | No |
|  | For the female groups, (1 – *Impact_HBonIIB_*), representing the impact of *Norm* on *IIB*, should be higher in the Moroccan than in the South-Asian Surinamese group. | Yes | Yes | Yes |
|  | For the Dutch group, (1 – *Impact_HBonIIB_*), representing the impact of *Norm* on *IIB*, should be higher in the female than in the male group. | No | No | No |
|  | For the South-Asian Surinamese group, (1 – *Impact_HBonIIB_*), representing the impact of *Norm* on *IIB*, should be higher in the female than in male group. | No | No | No |
|  | For the Moroccan group, (1 – *Impact_HBonIIB_*), representing the impact of *Norm* on *IIB*, should be higher in the female than in the male group. | No | No | No |
|  | For the male groups, *Intent_PAB_* should be higher in the Dutch than in the Moroccan group. | No | No | No |
|  | For the male groups, *Intent_PAB_* should be higher in the Dutch than in the South-Asian Surinamese group. | Yes | Yes | Yes |
|  | For the female groups, *Intent_PAB_* should be higher in the Dutch than in the Moroccan group. | No | No | No |
|  | For the female groups, *Intent_PAB_* should be higher in the Dutch than in the South-Asian Surinamese group. | Yes | Yes | Yes |
|  | For the Dutch group, *Intent_PAB_* should be higher in the female than in the male group. | Yes | Yes | Yes |
|  | For the South-Asian Surinamese group, *Intent_PAB_* should be higher in the male than in the female group. | No | No | No |
|  | For the Moroccan group, *Intent_PAB_* should be higher in the male than in the female group. | No | No | No |
|  | For the male groups, *Intent_EB_* should be higher in the Dutch than in the Moroccan group. | No | No | No |
|  | For the male groups, *Intent_EB_* should be higher in the Dutch than in the South-Asian Surinamese group. | Yes | Yes | Yes |
|  | For the female groups, *Intent_EB_* should be higher in the Dutch than in the Moroccan group. | No | No | No |
|  | For the female groups, *Intent_EB_* should be higher in the Dutch than in the South-Asian Surinamese group. | Yes | Yes | Yes |
|  | For the Dutch group, *Intent_EB_* should be higher in the female than in the male group. | Yes | Yes | Yes |
|  | For the Moroccan group, *Intent_EB_* should be higher in the female than in the male group. | Yes | Yes | Yes |
|  | For the South-Asian Surinamese group, *Intent_EB_* should be higher in the female than in the male group. | Yes | Yes | Yes |

# Results

## Optimisation

### Optimisation results

Table S8 shows the average optimal values for *Intent_EB_* and *Intent_PAB_* and the corresponding minimum cost value for 20 optimisation runs (using the basin-hopping algorithm (see ***Appendix 1.4***, subsection “**Basin-hopping algorithm**”) for each group, referring to the change that individuals make in eating behaviour and physical activity behaviour, respectively. Since each of the two sub-cost functions is standardised (see ***Appendix 1.4***, subsection “**Cost function**”), the minimum cost value can be 2. We also find that the results are the same for all 20 optimisation runs.

***Table S8:* Optimisation results including value for the cost function corresponding to the set of optimised parameters.**

| Socio-cultural group | *Intent_EB_* in (kcal/day)/(kg/m^2^) | *Intent_PAB_* in 1/(kg/m^2^) | Cost |
| --- | --- | --- | --- |
| Dutch men | -295.46 | 0.056 | 2.00 |
| Moroccan men | -301.31 | 0.056 | 2.00 |
| South-Asian Surinamese men | -283.19 | 0.055 | 2.00 |
| Dutch women | -251.46 | 0.060 | 2.00 |
| Moroccan women | -258.81 | 0.061 | 2.00 |
| South-Asian Surinamese women | -233.65 | 0.057 | 2.01 |

### Sensitivity analysis of *Intent_EB_* and *Intent_PAB_*

**Test 1: varying *Intent_EB_* and keeping *Intent_PAB_* fixed at its optimal value**

In this sensitivity analysis test we vary *Intent_EB_* from -50 to -1,200, whilst keeping *Intent_PAB_* fixed at its optimal value. The sensitivity analysis results are similar for all groups and therefore we show only the results for South-Asian Surinamese men and women, as they are representative for all groups. The results of this sensitivity analysis test (test 1) for South-Asian Surinamese men and women are shown in figures S6 and S7, respectively. From these figures we observe that as the value for *Intent_EB_* increases, the decrease in *MedBMI* gradually becomes sharp and that *MedBMI* reaches its stable value faster. For *Intent_EB_* values smaller than -500, the system gradually becomes unstable and tends towards oscillatory behaviour. We conclude from this that the SDMs are robust against small changes in *Intent_EB_*.

**Test 2: varying *Intent_PAB_* and keeping *Intent_EB_* fixed at the optimal value**

In this sensitivity analysis test we vary *Intent_PAB_* from 0.01 to 0.5, whilst keeping *Intent_EB_* fixed at its optimal value. The sensitivity analysis results are again similar for all groups and therefore we show only the results for South-Asian Surinamese men and women, as they are representative for all groups. The results of this sensitivity analysis test (test 2) for South-Asian Surinamese men and women are shown in figures S8 and S9, respectively. From these figures we observe that as the value for *Intent_PAB_* increases, the decrease in *MedBMI* gradually becomes sharp and that *MedBMI* reaches its stable value faster. For *Intent_PAB_* values greater than 0.2, the system gradually becomes unstable and tends towards oscillatory behaviour. We conclude from this that the SDMs are robust against small changes in *Intent_PAB_*.

From figures S6-S9, we also observe that the red line, representing ‘what if’ group-level BMI were driven only by health awareness, becomes oscillatory quickly and that the oscillatory peaks are high. The reason behind this behaviour is that the value for *IIB* is constant in this scenario (as *Norm* does not play a role anymore). However, if *Intent_EB_* or *Intent_PAB_* has a very high value then the *MedBMI* change is large, and this makes *Discrepancy_BMIandIIB_* become alternatively highly positive and negative. So, we see this oscillating behaviour quickly with high oscillatory peaks in the scenario representing driven only by health awareness (red line). In the scenario representing driven only by norms (blue line), and in the scenario representing driven by health awareness and norms combined (green line), the oscillations start later, and the oscillatory peaks are small because the value for *IIB* also changes based on the change in *MedBMI* (which adjusts *Norm*).


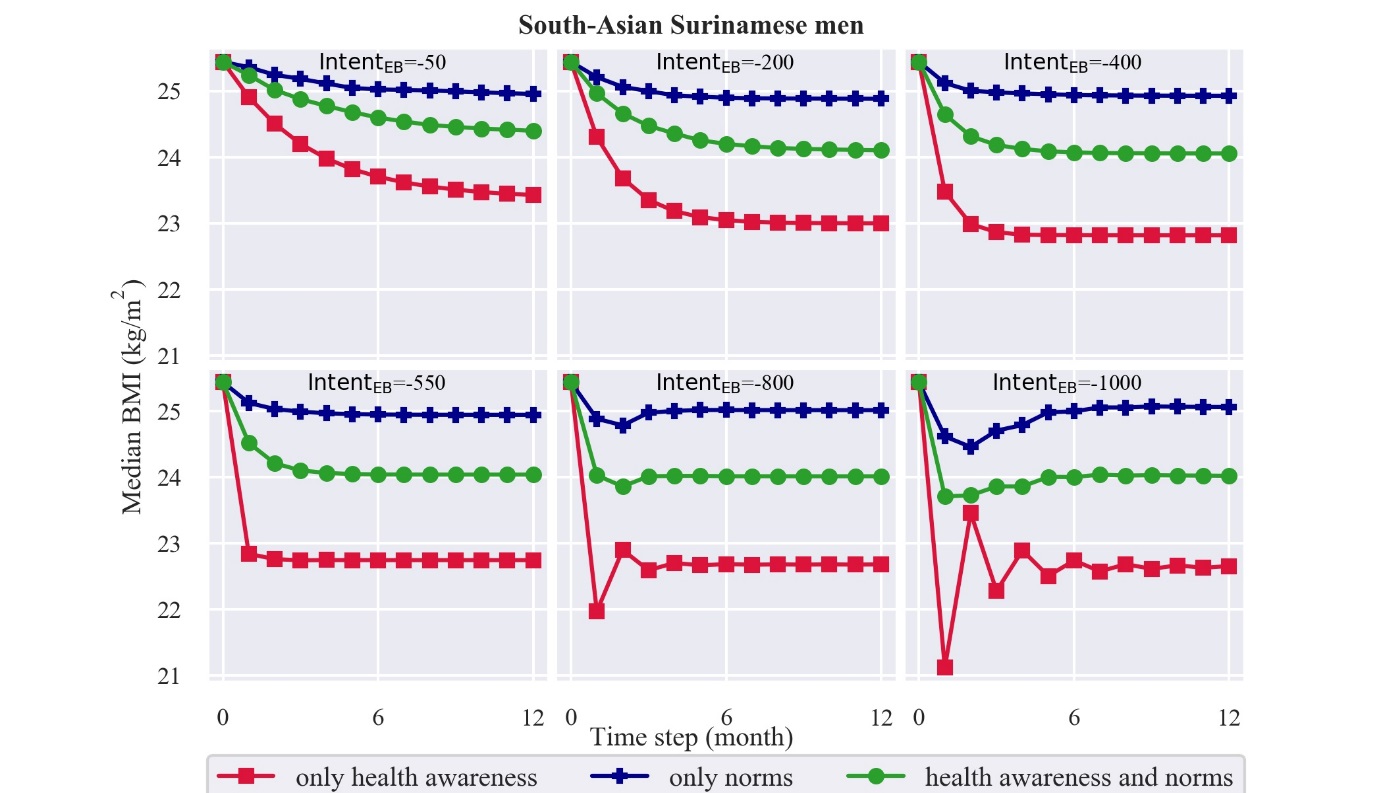


***Figure S6:* Test 1 of sensitivity analysis of *Intent_EB_* and *Intent_PAB_* on *MedBMI* for South-Asian Surinamese men. We vary *Intent_EB_* whilst keeping *Intent_PAB_* fixed at its optimal value.**


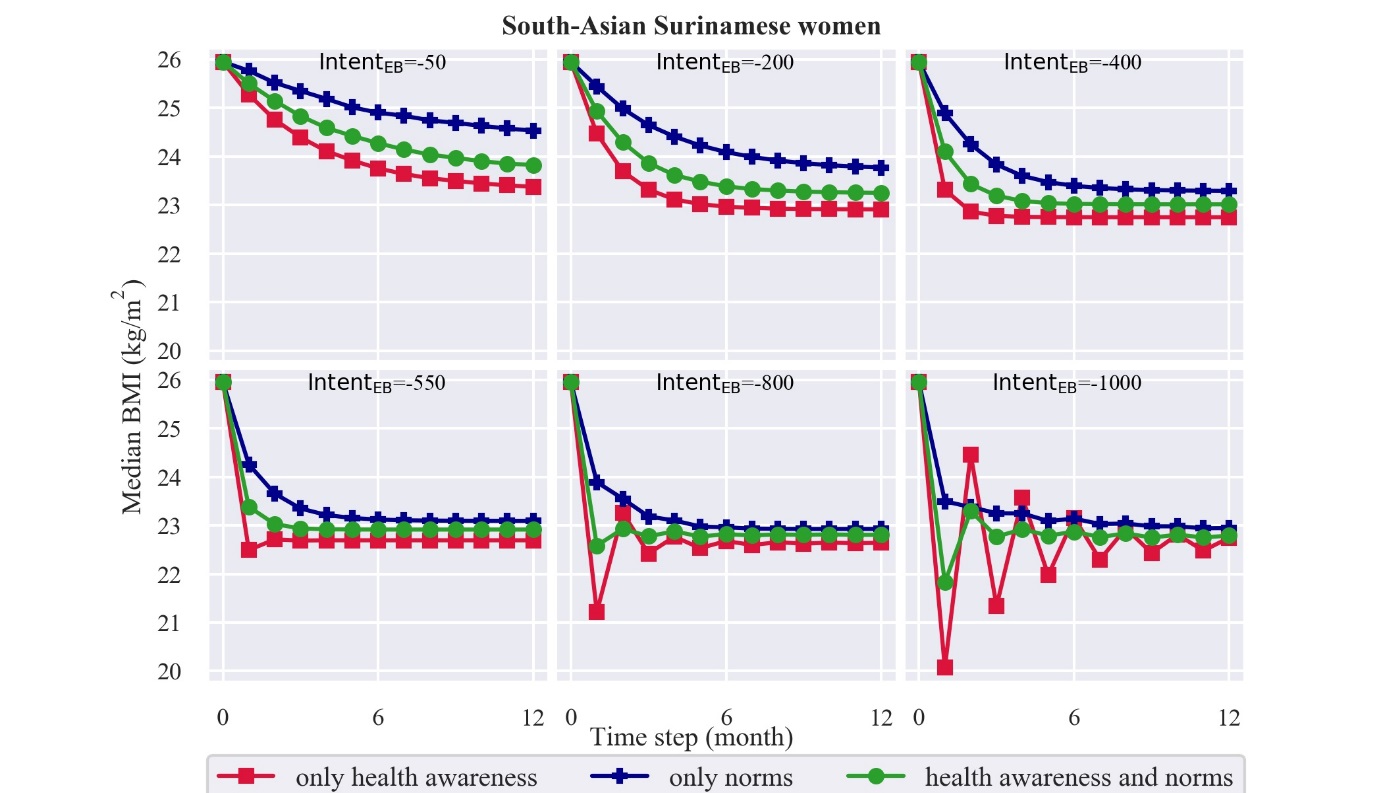


***Figure S7:* Test 1 of sensitivity analysis of *Intent_EB_* and *Intent_PAB_* on *MedBMI* for South-Asian Surinamese women. We vary *Intent_EB_* whilst keeping *Intent_PAB_* fixed at its optimal value.**

***
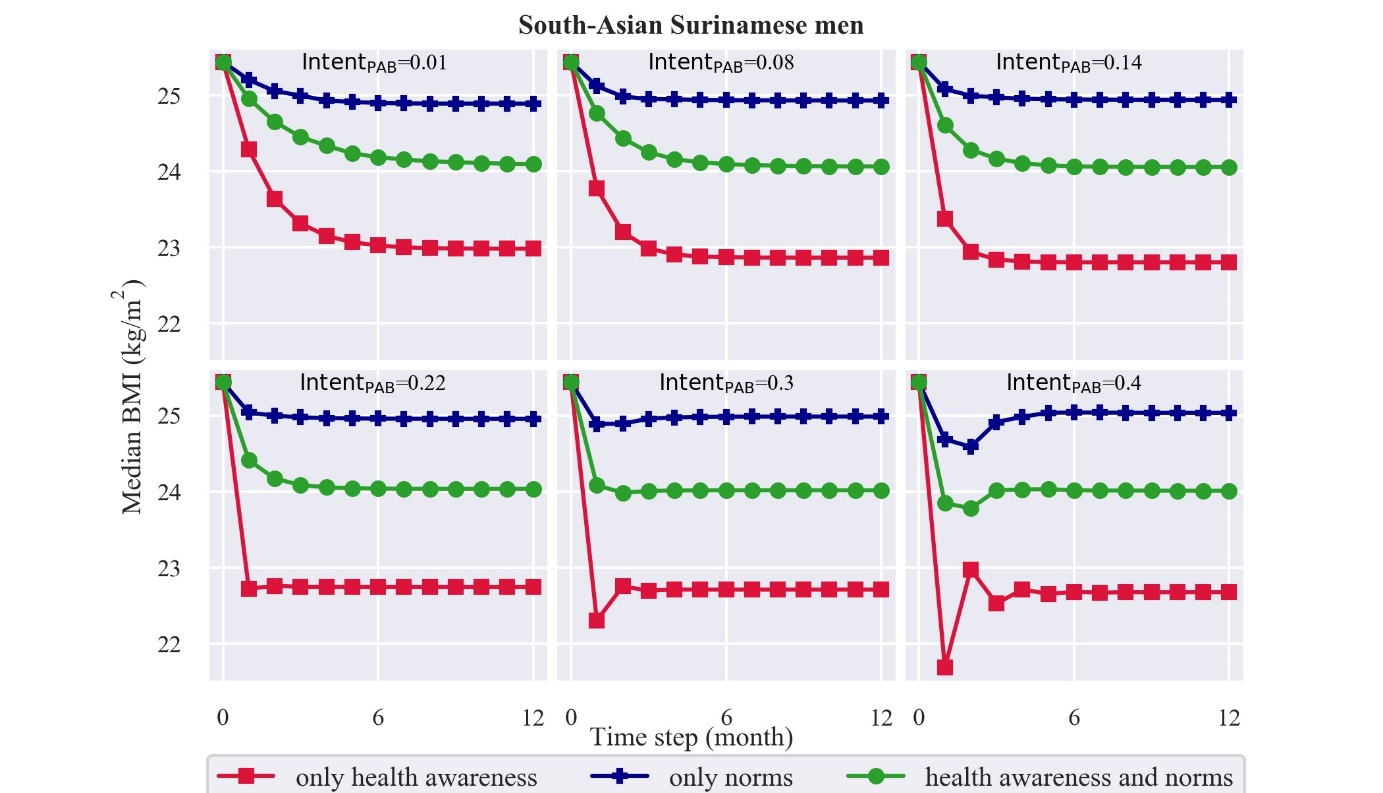
***

***Figure S8:* Test 2 of sensitivity analysis of *Intent_EB_* and *Intent_PAB_* on *MedBMI* for South-Asian Surinamese men. We vary *Intent_PAB_* whilst keeping *Intent_EB_* fixed at its optimal value.**


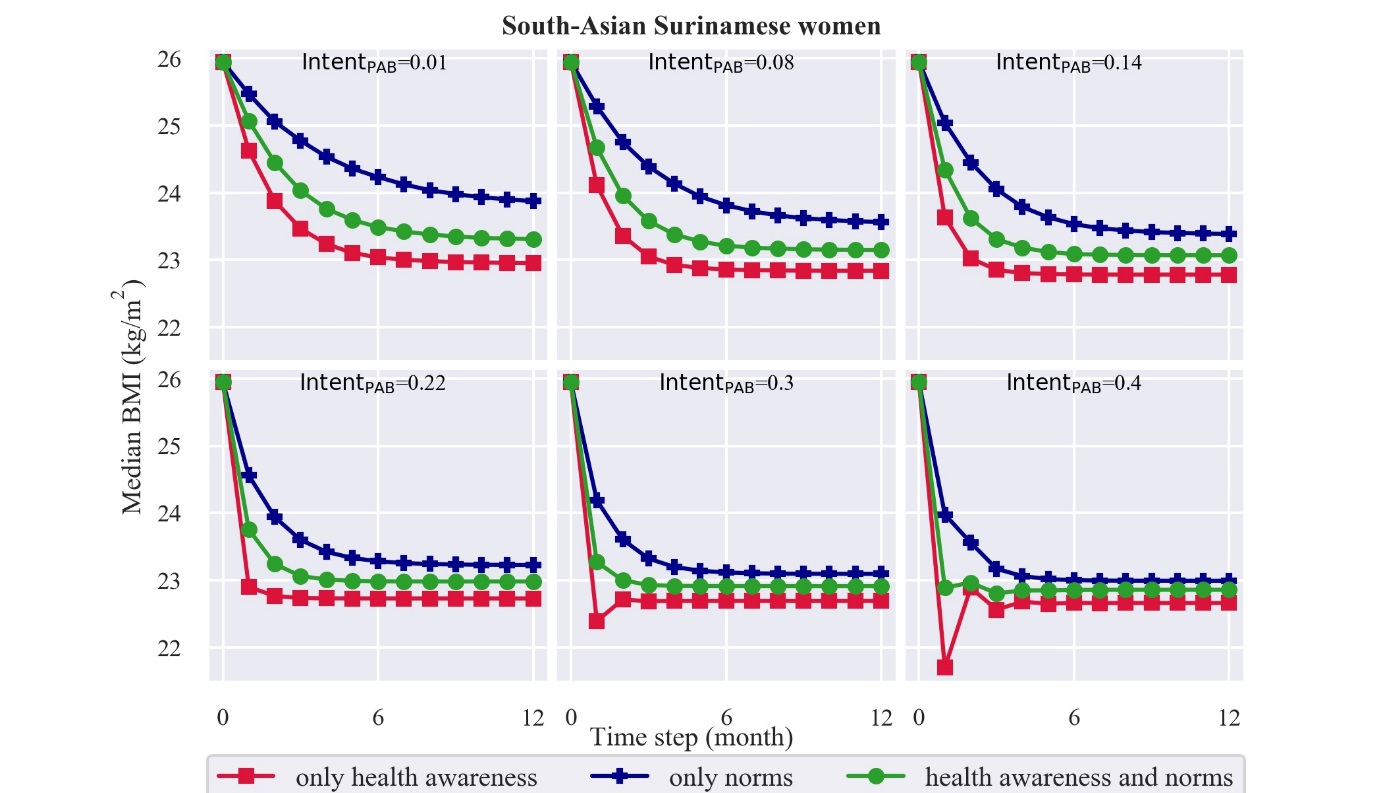


***Figure S9:* Test 1 of sensitivity analysis of *Intent_EB_* and *Intent_PAB_* on *MedBMI* for South-Asian Surinamese women. We vary *Intent_EB_* whilst keeping *Intent_PAB_* fixed at its optimal value.**

## Scenarios

### 95% confidence interval

We determine the confidence interval (CI) of the group-level BMI at each time step time for the three scenarios, shown in figure 6 in the main manuscript as the shaded area around the group-level BMI at each time step. Since the group-level BMI at each time step is the median of the individual values for *BMI* at that time step, we use the formula of CI for medians to determine the CIs at each time step. The lower 95% confidence limit is given by the value at rank:

$$\frac{n}{2}-\frac{1.96\sqrt{n}}{2}.$$

The upper 95% confidence limit is given by the value at rank:

$$1+\frac{n}{2}+\frac{1.96\sqrt{n}}{2}.$$

### Differences between socio-cultural groups in change in group-level BMI over time for scenarios

If driven by only health awareness, median BMI decreases by 10% (3 BMI points) among Dutch, 12% (3 points) among Moroccan, and 10% (3 points) among South-Asian Surinamese men. It drops 8% (2 points) among Dutch, 12% (3 points) among Moroccan, and 12% (3 points) among South-Asian Surinamese women.

If driven by only norms, there is a reduction in median BMI of 2% (1 point) among Dutch, 3% (1 point) among Moroccan, and 2% (1 point) among South-Asian Surinamese men. The drop is 7% (2 points) among Dutch, 8% (2 points) among Moroccan, and 9% (2 points) among South-Asian Surinamese women.

If driven by health awareness and norms, median BMI drops 5% (1 points) among Dutch, 5% (1 point) among Moroccan, and 5% (1 point) among South-Asian Surinamese men. It decreases by 8% (2 points) among Dutch, 11% (3 points) among Moroccan, and 11% (3 points) among South-Asian Surinamese women.

### Sensitivity analysis of *Impact_HBonIIB_*

To test how sensitive the change in *MedBMI* is to *Impact_HBonIIB_*, we increase and decrease the value of *Impact_HBonIIB_* by 50% and analyse the change in stable *MedBMI* at convergence as follows:

$$\Delta MedBMI=\frac{{MedBMI}_{altered ImpactHBonIIB}\left( stable \right)-{MedBMI}_{original ImpactHBonIIB}(stable)}{{MedBMI}_{original ImpactHBonIIB}(stable)}\times100,$$

where ${MedBMI}_{altered ImpactHBonIIB}\left( stable \right)$ is the stable *MedBMI* with the 50% altered *Impact_HBonIIB_* and ${MedBMI}_{original ImpactHBonIIB}(stable)$ is the stable *MedBMI* with the original *Impact_HBonIIB_*. From the results given in table S9, we observe that the percentage change in stable *MedBMI* for a 50% change in *Impact_HBonIIB_* is very small (<2%). We conclude that *MedBMI* is not sensitive to changes in *Impact_HBonIIB_*.

***Table S9:* Sensitivity analysis of *Impact_HBonIIB_*, showing the percentage change in stable *MedBMI* for a 50% increase and decrease in the value for *Impact_HBonIIB_*.**

| Socio-cultural group | Δ*MedBMI*  (50% increase in *Impact_HBonIIB_*), % | Δ*MedBMI*  (50% decrease in *Impact_HBonIIB_*), % |
| --- | --- | --- |
| Dutch men | -1.13 | 0.16 |
| Moroccan men | -1.19 | 1.37 |
| South-Asian Surinamese men | -1.30 | 1.62 |
| Dutch women | -0.18 | 0.16 |
| Moroccan women | -0.85 | 1.09 |
| South-Asian Surinamese women | -0.75 | 0.79 |

# References

1. Snijder MB, Galenkamp H, Prins M, et al. Cohort profile: The Healthy Life in an Urban Setting (HELIUS) study in Amsterdam, the Netherlands. *BMJ Open*. 2017;7(12):1-11. doi:10.1136/bmjopen-2017-017873

2. Stronks K, Snijder MB, Peters RJ, Prins M, Schene AH, Zwinderman AH. Unravelling the impact of ethnicity on health in Europe: the HELIUS study. *BMC Public Health*. 2013;13(1):402. doi:10.1186/1471-2458-13-402

3. FAO, WHO, UNU. *Human Energy Requirements*. Rome; 2001.

4. Pulvers KM, Lee RE, Kaur H, et al. Development of a culturally relevant body image instrument among urban African Americans. *Obes Res*. 2004;12(10):1641-1651. http://ovidsp.ovid.com/ovidweb.cgi?T=JS&PAGE=reference&D=emed9&NEWS=N&AN=39707006.

5. Nicolaou M, Gademan MGJ, Snijder MB, et al. Validation of the SQUASH Physical Activity Questionnaire in a Multi-Ethnic Population: The HELIUS Study. Buchowski M, ed. *PLoS One*. 2016;11(8):e0161066. doi:10.1371/journal.pone.0161066

6. Murakami K, Livingstone MBE. Prevalence and characteristics of misreporting of energy intake in US adults: NHANES 2003–2012. *Br J Nutr*. 2015;114(8):1294-1303. doi:10.1017/S0007114515002706

7. Hill JO, Catenacci VA, Wyatt HR. Obesity: Etiology. In: Shils ME, Shike M, Ross AC, Caballero B, Cousins RJ, eds. *Modern Nutrition in Health and Disease*. 10th ed. Lippincott Williams & Wilkins; 2006:1019.

8. Katan MB, Ludwig DS. Extra Calories Cause Weight Gain — But How Much? *JAMA*. 2010;303(1):65-66. doi:10.3945/ajcn.2009.28595.9.

9. Peeling E, Tucker A. Making Time: Pseudo Time-Series for the Temporal Analysis of Cross Section Data. In: Berthold MR, Shawe-Taylor J, Lavrac N, eds. *Advances in Intelligent Data Analysis VII - 7th International Symposium on Intelligent Data Analysis, IDA 2007*. Ljubljana, Slovenia; 2007:184-194.

10. Tucker A, Garway-Heath D. The Pseudotemporal Bootstrap for Predicting Glaucoma From Cross-Sectional Visual Field Data. *IEEE Trans Inf Technol Biomed*. 2010;14(1):79-85. doi:10.1109/TITB.2009.2023319

11. Li Y, Swift S, Tucker A. Modelling and analysing the dynamics of disease progression from cross-sectional studies. *J Biomed Inform*. 2013;46(2):266-274. doi:10.1016/j.jbi.2012.11.003

12. Wales DJ, Doye JPK. Global Optimization by Basin-Hopping and the Lowest Energy Structures of Lennard-Jones Clusters Containing up to 110 Atoms. *J Phys Chem A*. 1997;101(28):5111-5116. doi:10.1021/jp970984n

13. SciPy v1.0.0 Reference Guide. scipy.optimize.basinhopping. https://docs.scipy.org/doc/scipy/reference/generated/scipy.optimize.basinhopping.html#scipy.optimize.basinhopping. Accessed December 5, 2019.

14. Goldstein H, Poole C, Safko J. *Classical Mechanics*. 3rd ed. Addison Wesley; 2001.

15. Taherian Fard A, Ragan MA. Modeling the Attractor Landscape of Disease Progression: a Network-Based Approach. *Front Genet*. 2017;8(APR):1-11. doi:10.3389/fgene.2017.00048

16. Boelkins M. Active Calculus - single variable. https://activecalculus.org/single/sec-1-8-tan-line-approx.html. Published 2018. Accessed June 6, 2019.

17. Weinsier RL, Wilson LJ, Lee J. Medically safe rate of weight loss for the treatment of obesity: A guideline based on risk of gallstone formation. *Am J Med*. 1995;98(2):115-117. doi:10.1016/S0002-9343(99)80394-5

18. National Institutes of Health. Clinical Guidelines on the Identification, Evaluation, and Treatment of Overweight and Obesity in Adults--The Evidence Report. National Institutes of Health. *Obes Res*. 1998;6 Suppl 2(September):51S-209S. http://www.ncbi.nlm.nih.gov/pubmed/9813653.

19. McAuley PA, Chen H, Lee D, Artero EG, Bluemke DA, Burke GL. Physical Activity, Measures of Obesity, and Cardiometabolic Risk: The Multi-Ethnic Study of Atherosclerosis (MESA). *J Phys Act Heal*. 2014;11(4):831-837. doi:10.1123/jpah.2012-0068a

20. Burke GL, Bertoni AG, Shea S, et al. The Impact of Obesity on Cardiovascular Disease Risk Factors and Subclinical Vascular Disease: The Multi-Ethnic Study of Atherosclerosis. *Arch Intern Med*. 2008;168(9):928. doi:10.1001/archinte.168.9.928

21. Brouwer L, Lalmahomed B, Josias H. *Andere Tijden, Andere Meiden: Een Onderzoek Naar Het Weglopen van Marokkaanse, Turkse, Hindostaanse En Creoolse Meisjes [Different Times, Different Girls: A Study of Moroccan, Turkish, Hindustani and Creole Runaway Girls]*. Utrecht, The Netherlands: Van Arkel; 1992.

22. Dekovic M, Pels T, Model S. *Child Rearing in Six Ethnic Families: The Multi-Cultural Dutch Experience*. Lewiston, N.Y: Edwin Mellen Press; 2006.

23. Mungra G. Hindoestaanse gezinnen in Nederland. 1990.

24. van Bergen DD, van Balkom AJLM, Smit JH, Saharso S. “ I felt so hurt and lonely ”: Suicidal behavior in South Asian-Surinamese, Turkish, and Moroccan women in the Netherlands. *Transcult Psychiatry*. 2012;49(1):69-86. doi:10.1177/1363461511427353

25. Jhangiani R, Tarry H, Stangor C. Person, Gender, and Cultural Differences in Conformity. In: *Principles of Social Psychology – 1st International Edition*. Victoria, B.C: BCcampus; 2014:328-336.

26. Gibson-Smith D, Bot M, Snijder M, et al. The relation between obesity and depressed mood in a multi-ethnic population. The HELIUS study. *Soc Psychiatry Psychiatr Epidemiol*. 2018;53(6):629-638. doi:10.1007/s00127-018-1512-3

27. Ikram UZ, Snijder MB, de Wit MAS, Schene AH, Stronks K, Kunst AE. Perceived ethnic discrimination and depressive symptoms: the buffering effects of ethnic identity, religion and ethnic social network. *Soc Psychiatry Psychiatr Epidemiol*. 2016;51(5):679-688. doi:10.1007/s00127-016-1186-7

28. Osei-Kwasi HA, Nicolaou M, Powell K, et al. Systematic mapping review of the factors influencing dietary behaviour in ethnic minority groups living in Europe: a DEDIPAC study. *Int J Behav Nutr Phys Act*. 2016;13(1):17. doi:10.1186/s12966-016-0412-8

29. Hosper K, Deutekom M, Stronks PK. The effectiveness of “Exercise on Prescription” in stimulating physical activity among women in ethnic minority groups in the Netherlands: protocol for a randomized controlled trial. *BMC Public Health*. 2008;8(406):8. doi:10.1186/1471-2458-8-406

30. Kahan D. Adult physical inactivity prevalence in the Muslim world: Analysis of 38 countries. *Prev Med Reports*. 2015;2:71-75. doi:10.1016/j.pmedr.2014.12.007

31. Sharara E, Akik C, Ghattas H, Makhlouf Obermeyer C. Physical inactivity, gender and culture in Arab countries: a systematic assessment of the literature. *BMC Public Health*. 2018;18(639):19. doi:10.1186/s12889-018-5472-z

32. Rhodes RE, Macdonald HM, McKay HA. Predicting physical activity intention and behaviour among children in a longitudinal sample. *Soc Sci Med*. 2006;62(12):3146-3156. doi:10.1016/j.socscimed.2005.11.051

33. Hendriks A-M, Gubbels JS, Jansen MWJ, Kremers SPJ. Health Beliefs regarding Dietary Behavior and Physical Activity of Surinamese Immigrants of Indian Descent in The Netherlands: A Qualitative Study. *ISRN Obes*. 2012;2012:1-8. doi:10.5402/2012/903868

34. Nicolaou M, Vlaar E, van Valkengoed I, Middelkoop B, Stronks K, Nierkens V. Development of a diabetes prevention program for Surinamese South Asians in the Netherlands. *Health Promot Int*. 2014;29(4):680-691. doi:10.1093/heapro/dat018

35. Nicolaou M, Benjelloun S, Stronks K, van Dam RM, Seidell JC, Doak CM. Influences on body weight of female Moroccan migrants in the Netherlands: A qualitative study. *Health Place*. 2012;18(4):883-891. doi:10.1016/j.healthplace.2012.03.001

36. World Health Organization. WHO Global Health Observatory Data Repository [online database]. http://apps.who.int/gho/data/view.main. Published 2013. Accessed May 21, 2013.

37. World Health Organization. *Country Profiles on Nutrition, Physical Activity and Obesity in the 53 WHO European Region Member States: Netherlands*.; 2013. http://www.euro.who.int/__data/assets/pdf_file/0018/243315/Netherlands-WHO-Country-Profile.pdf. Accessed June 5, 2019.

38. Gijsberts M, Dagevos J. *Jaarrapport Integratie 2009*. Den Haag: Sociaal en Cultureel Planbureau; 2009.

39. Lawton J, Ahmad N, Hanna L, Douglas M, Hallowell N. ‘I can’t do any serious exercise’: barriers to physical activity amongst people of Pakistani and Indian origin with Type 2 diabetes. *Health Educ Res*. 2006;21(1):43-54. doi:10.1093/her/cyh042

40. Visram S, Crosland A, Unsworth J, Long S. Engaging women from South Asian communities in cardiac rehabilitation. *Int J Ther Rehabil*. 2008;15(7):298-305.

41. Sriskantharajah J, Kai J. Promoting physical activity among South Asian women with coronary heart disease and diabetes: what might help? *Fam Pract*. 2006;24(1):71-76. doi:10.1093/fampra/cml066

42. Khanam S, Costarelli V. Attitudes towards health and exercise of overweight women. *J R Soc Promot Health*. 2008;128(1):26-30. doi:10.1177/1466424007085225

43. Abbott S, Riga M. Delivering services to the Bangladeshi community: the views of healthcare professionals in East London. *Public Health*. 2007;121(12):935-941. doi:10.1016/j.puhe.2007.04.014

44. Carroll R, Ali N, Azam N. Promoting physical activity in South Asian Muslim women through “exercise on prescription.” *Health Technol Assess (Rockv)*. 2002;6(8):6-9. doi:10.3310/hta6080

45. Patel M, Phillips-Caesar E, Boutin-Foster C. Barriers to Lifestyle Behavioral Change in Migrant South Asian Populations. *J Immigr Minor Heal*. 2012;14(5):774-785. doi:10.1007/s10903-011-9550-x

46. Baddou I, El Hamdouchi A, El Harchaoui I, et al. Objectively Measured Physical Activity and Sedentary Time among Children and Adolescents in Morocco: A Cross-Sectional Study. *Biomed Res Int*. 2018;2018:1-7. doi:10.1155/2018/8949757

47. Batnitzky A. Obesity and household roles: gender and social class in Morocco. *Sociol Health Illn*. 2008;30(3):445-462. doi:10.1111/j.1467-9566.2007.01067.x

48. Crawford R. Risk Ritual and the Management of Control and Anxiety in Medical Culture. *Heal An Interdiscip J Soc Study Heal Illn Med*. 2004;8(4):505-528. doi:10.1177/1363459304045701

49. Dean K. Self-care components of lifestyles: The importance of gender, attitudes and the social situation. *Soc Sci Med*. 1989;29(2):137-152. doi:10.1016/0277-9536(89)90162-7

50. Teuscher D, Bukman AJ, van Baak MA, Feskens EJM, Renes RJ, Meershoek A. Challenges of a healthy lifestyle for socially disadvantaged people of Dutch, Moroccan and Turkish origin in the Netherlands: a focus group study. *Crit Public Health*. 2015;25(5):615-626. doi:10.1080/09581596.2014.962013

51. Nicolaou M, Doak CM, van Dam RM, Brug J, Stronks K, Seidell JC. Cultural and Social Influences on Food Consumption in Dutch Residents of Turkish and Moroccan Origin: A Qualitative Study. *J Nutr Educ Behav*. 2009;41(4):232-241. doi:10.1016/j.jneb.2008.05.011

52. Blokstra A, Burns C, Seidell J. Perception of weight status and dieting behaviour in Dutch men and women. *Int J Obes*. 1999;23(1):7-17. doi:10.1038/sj.ijo.0800803

53. Nicolaou M, Palsma AH, Stronks K. *De Voeding van Jonge Volwassenen van Turkse En Marokkaanse Afkomst: ‘Dietary Habits among Young Dutch People of Turkish and Moroccan Origin: A Problem Analysis and Needs Assessment for the Development of Appropriate Nutrition Interventions.’* Amsterdam; 2007.

54. U.S. Department of Health and Human Services and U.S. Department of Agriculture. *2015–2020 Dietary Guidelines for Americans*. 8th ed.; 2015. doi:10.1097/NT.0b013e31826c50af

55. NHS UK. What should my daily intake of calories be? https://www.nhs.uk/common-health-questions/food-and-diet/what-should-my-daily-intake-of-calories-be/. Published 2016. Accessed June 5, 2019.

56. Voedingscentrum. Energie (calorieën). https://www.voedingscentrum.nl/encyclopedie/energie.aspx. Published 2019. Accessed June 5, 2019.

57. Centraal Bureau voor de Statistiek (CBS). *Bevolking; Leeftijd, Migratieachtergrond, Geslacht En Regio, 1 Januari*. The Netherlands; 2019. https://opendata.cbs.nl/statline/#/CBS/nl/dataset/37713/table?dl=27C40.

58. Gemeente Amsterdam. *2.1.11 Bevolking Naar Leeftijdsgroepen En Migratieachtergrond, 1 Januari 2019*.; 2019. https://data.amsterdam.nl/datasets/kKtqkkhpvLUXsg/bevolking-stand-van-de-bevolking-amsterdam/.
